# Numb‐Associated Kinases‐Mediated AP2M1 Activation Facilitates Porcine Reproductive and Respiratory Syndrome Virus Entry

**DOI:** 10.1155/tbed/1766395

**Published:** 2026-04-13

**Authors:** Longxiang Zhang, Rui Li, Lingqiao You, Yan Jiang, Xinrong Wang, Junhai Zhu, Nan Yan, Yue Wang

**Affiliations:** ^1^ College of Veterinary Medicine, Southwest University, Chongqing, 400715, China, swu.edu.cn; ^2^ National Center of Technology Innovation for Pigs, Chongqing, 402460, China

**Keywords:** AAK1, AP2M1, BMP2K, entry, GAK, PRRSV

## Abstract

Porcine reproductive and respiratory syndrome virus (PRRSV) is a major pathogen that poses a considerable threat to the global swine industry, particularly with emerging variants that complicate control efforts. However, the host factors involved in PRRSV entry are not well understood. In the present study, we identified members of the numb‐associated kinase (NAK) family, specifically adaptor‐associated kinase 1 (AAK1), G‐associated kinase (GAK), and BMP‐2‐inducible kinase (BMP2K), as essential regulators of PRRSV entry utilizing both genetic and pharmacological approaches. Mechanistically, NAKs facilitate PRRSV entry by phosphorylating the adaptor protein complex 2 subunit mu‐1 (AP2M1) at threonine 156, enhancing AP2M1 activation and thereby promoting its interaction with the YxxØ motif in PRRSV glycoprotein (GP) 5 and the receptor cluster of differentiation 163 (CD163). This interaction is critical for efficient trafficking of the virions to early endosomes (EEs). Disruption of AP2M1 phosphorylation or blockade of the AP2M1–YxxØ interaction significantly impaired PRRSV internalization, indicating the potential for targeting this pathway to inhibit infection. Notably, inhibition of the NAKs–AP2M1 axis effectively reduced infection across multiple PRRSV strains, highlighting its capacity as a broad‐spectrum antiviral target. Collectively, our findings provide novel insights into PRRSV entry mechanisms and offer a promising therapeutic strategy to control emerging variants of this economically significant disease.

## 1. Introduction

Porcine reproductive and respiratory syndrome (PRRS) is a highly contagious viral disease leading to reproductive failures in late‐term gestating sows and severe respiratory distress across all age groups of pigs [1, 2]. Despite its prolonged presence, controlling PRRS effectively continues to pose significant challenges [3]. The etiological agent, PRRS virus (PRRSV), is an enveloped, single‐stranded positive‐sense RNA virus classified within the order Nidovirales, family Arteriviridae, and genus *Betaarterivirus* [4]. The PRRSV genome (~15 kb) encodes two large replicase polyproteins and eight functionally distinct structural proteins: glycoprotein (GP) 2, 3, 4, 5, and 5a, along with the envelope protein, matrix protein, and nucleocapsid (N) proteins [5, 6].

The PRRSV life cycle comprises several distinct stages: attachment, entry, replication, assembly, and release [7]. The infection is initiated when the viral particles attach to the host cell surface, primarily through interacting with heparan sulfate, then binding to sialoadhesin and subsequent internalization predominantly via clathrin‐mediated endocytosis (CME) for delivery into early endosomes (EEs) [8–10]. Within these endosomes, PRRSV GPs interact with the key entry receptor, cluster of differentiation 163 (CD163), which triggers membrane fusion and uncoating [11–13]. Subsequently, the PRRSV genome is released from the nucleocapsid into the cytoplasm, where viral replication is completed [7, 14]. Newly synthesized viral components are assembled and enveloped within the endoplasmic reticulum (ER) and/or Golgi apparatus (GA) compartments, and the mature progeny virions are ultimately released to infect the neighboring cells via the exocytosis pathway [15–17]. Although previous studies have established a comprehensive framework for the PRRSV life cycle, research on the cellular factors involved in viral entry remains limited, thereby impeding the development of targeted antiviral strategies.

The numb‐associated kinase (NAK) family, which comprises adaptor‐associated kinase 1 (AAK1), cyclin G‐associated kinase (GAK), BMP‐2‐inducible kinase (BMP2K), and serine/threonine kinase 16 (STK16), plays pivotal roles in various cellular functions, including endocytosis, autophagy, signal transduction, and cell cycle regulation [18]. These kinases share limited sequence homology within their kinase domains and exhibit low similarity in other regions of the proteins [19]. Notably, STK16 is the most recently identified member of this family [20]. AAK1, GAK, and BMP2K contribute to CME by phosphorylating the threonine residue at position 156 (T156) of the adaptor protein complex 2 subunit mu‐1 (AP2M1) [21–23]. AP2M1 is an integral component of the AP2 heterotetrameric complex and works with other subunits (α, β, and σ) in the CME pathway [24, 25]. As a pivotal initiator of CME, AP2M1 recognizes the YxxØ motif (where the “x” represents any amino acid and the “Ø” denotes hydrophobic residues such as L/M/F/I/V) in cargo proteins through its hydrophobic pocket, thereby facilitating the incorporation of these proteins into clathrin‐coated vesicles and ultimately mediating their intracellular trafficking [26–29]. Previous research has identified NAKs as potential broad‐spectrum antiviral targets due to their crucial roles in the infection of multiple viruses [30–33]. However, the precise roles of NAKs in PRRSV infection have not been elucidated.

In this study, we first identified that members of the NAK family, specifically AAK1, GAK, and BMP2K, but not STK16, were required for PRRSV entry. Furthermore, we demonstrated that NAKs regulated PRRSV entry by phosphorylating the adaptor protein AP2M1, which interacted with viral GP5 and its receptor CD163 through the conserved YxxØ motif.

## 2. Materials and Methods

### 2.1. Cells and Viruses

Marc145 and HEK‐293T cell lines were cultured in Dulbecco’s modified eagle medium (DMEM; #12100; Solarbio) supplemented with 10% fetal bovine serum (FBS; #AFD050; Clarkbio). The PRRSV‐susceptible immortalized porcine alveolar macrophages (iPAMs), established through stable expression of the SV40 large T antigen, were generously provided by Y.‐D. Tang et al. [34] (Harbin Veterinary Research Institute). The iPAM cells were cultured in RPMI‐1640 (#C11875500BT; Gibco) containing 10% FBS and 1% antibiotics.

PRRSV strains HN07‐1 (GenBank: KX766378.1, highly pathogenic [HP]) and HNhx (GenBank: KX766379.1, NADC30‐like) were kindly provided by researcher Songlin Qiao from Henan Academy of Agricultural Sciences. BJ‐4 (GenBank: AF331831.1, classical), HuN4 (GenBank: EF635006, HP), and modified live vaccine (MLV; GenBank: AF159149, North American VR2332) were maintained in our laboratory. Unless stated otherwise, the BJ‐4 strain was used as the primary strain for all experimental procedures.

### 2.2. Antibodies

Mouse anti‐Flag monoclonal antibody (MAb; #MA1‐91878), goat anti‐mouse IgG polyclonal antibodies (pAbs; #G‐21040), and goat anti‐rabbit IgG pAbs (#G‐21234) were obtained from Invitrogen. Mouse anti‐β‐actin MAb (#A2228) was obtained from Sigma‐Aldrich. Rabbit anti‐EEA1 pAbs (#68065‐1‐Ig) and rabbit anti‐CD163 pAbs (#16646‐1‐AP) were obtained from Proteintech. Rabbit anti‐AP2M1 pAbs (#AB75995) and rabbit anti‐AP2M1[T156] pAbs (#AB109397) were obtained from Abcam. Mouse anti‐CD163 MAb (#GTX42364) and rabbit anti‐PRRSV N pAbs (#GTX129270) were obtained from GeneTex. Rabbit anti‐Myc pAbs (#ABT2060) was obtained from Abkine. AF555‐donkey anti‐rabbit IgG (#A0468), AF647‐Goat anti‐rabbit IgG (#A0428), and AF488‐goat anti‐mouse IgG (#A0428) were obtained from Beyotime. Mouse anti‐PRRSV N or GP5 MAb was developed in our lab.

### 2.3. Expression Vector Construction and Transfection

The pFlag‐GP5 and pFlag‐CD163 plasmids were constructed and maintained in our laboratory. The complementary DNA (cDNA) of GP5 was amplified from the PRRSV strain BJ‐4 genome utilizing the PrimeSTAR HS DNA polymerase (#R010A; TaKaRa) and subsequently inserted into the pCAGGS vector with a Flag tag. Similarly, the CD163 cDNA was extracted from iPAM cells and inserted into the pCAGGS‐Flag vector. The pCMV‐3×Myc‐AP2M1 (#P58817) was obtained from Miaoling Biotechnology. The pEGFP‐AP2M1, pEGFP‐AP2M1[T156A], and pmCherry‐GP5 constructs were synthesized by Sangon Biotech. These vectors were transfected into the designated cells using the Lipofectamine 2000 or Lipofectamine LTX & Plus reagent.

### 2.4. Small‐Interference RNA (siRNA) Assay

The siRNAs specific to NAKs, including GAK, AAK1, BMP2K, and STK16, as well as AP2M1, were created and produced by GenePharma. Cells underwent transfection with the specified siRNAs at a final concentration of 50 nM using siRNA‐Mate Plus reagent (#G04026; GenePharma). The knockdown efficiency of the above‐mentioned siRNAs was evaluated through quantitative real‐time PCR (RT‐qPCR) and/or western blotting (WB). The specific siRNAs were listed in Table 1.

**Table 1:** siRNAs used in this study.

Target genes	5^’^–3^’^ (sense)	5^’^–3^’^ (antisense)
Monkey‐AAK1	GCUCUCAACAGCAACUAAUTT	AUUAGUUGCUGUUGAGAGCTT
Monkey‐GAK	GUGACAUUCAAGAGAAAUATT	UAUUUCUCUUGAAUGUCACTT
Monkey‐BMP2K	GAGAGUCAGGUUGCUAUCUTT	AGAUAGCAACCUGACUCUCTT
Monkey‐STK16	GGGUUACAUGAUGGACACUTT	AGUGUCCAUCAUGUAACCCTT
Monkey/pig‐AP2M1	GGAAGCAAUCAAUUGCCAUTT	AUGGCAAUUGAUUGCUUCCTT
siNC	UUCUCCGAACGUGUCACGUTT	ACGUGACACGUUCGGAGAATT

### 2.5. Cell Viability Assay

The cells were either transfected with siRNAs targeting NAKs and AP2M1 or treated with varying concentrations of inhibitors, including sunitinib (#HY‐10255A; MedChemExpress), erlotinib (#HY‐50896; MedChemExpress), and N‐(p‐amylcinnamoyl)anthranilic acid (ACA; #HY‐118628; MedChemExpress), all of which were dissolved in DMSO with a final concentration of 0.02% (v/v). Cell viability was assessed using the enhanced cell counting kit‐8 (CCK‐8; #C6005; NCM Biotech). Briefly, a 20 μL aliquot of CCK‐8 solution was added to the cells with siRNA transfection or inhibitor treatment, followed by an incubation period for 1–4 h. The absorbance was then measured at 490 nm using a Multiskan FC microplate reader (Thermo Fisher Scientific).

### 2.6. RT‐qPCR

Total RNA was isolated from cells using the RNA extraction kits (#BSC52; BioFlux), followed by reverse transcription into cDNA with PrimeScript RT Master Mix (#RR036A; Takara). RT‐qPCR was performed on a Gentier 96E/R detection system (TIANLONG) with ChamQ Universal SYBR qPCR Master Mix (#Q711‐02; Vazyme). Relative gene expression level was calculated using the 2^−ΔΔCT^ method, with GAPDH as the endogenous control. The primer sequences were listed in Table 2.

**Table 2:** Primers for RT‐qPCR used in this study.

Target genes	Sequence (5^’^–3^’^)	
	Forward	Reverse
GAPDH	CCTTCCGTGTCCCTACTGCCAAC	GACGCCTGCTTCACCACCTTCT
PRRSV‐ORF7	AAACCAGTCCAGAGGCAAGG	GCAAACTAAACTCCACAGTGTAA
AAK1	GTCACAGTGGACGAGGTGTT	CCCTGACAGGTCCCTCATTA
GAK	TGGCCGAAGGAGGGTTT	AACTGTACGATGTTCGGGTG
BMP2K	TCCCAGTGTGCTGACCATTC	TTCAGGGCCATTTCCAGGTC
STK16	CCCTGAAGCGAATCCTGTGT	CCCTCAGACAGTAAGCCACG
AP2M1	AAACAAGCAAGAGTGGGAAGC	CATCTGGCGGGATAAAGC

### 2.7. WB

The cells were rinsed with PBS and then lysed in RIPA lysis buffer (#P0013B; Beyotime). The cell lysates were kept on ice for 30 min before centrifugation at 12,000 × *g* at 4°C for 15 min. Protein samples were loaded onto 10% or 12% SDS–PAGE gels for separation. Following transfer onto PVDF membranes (Merck Millipore, #03010040001), the membranes were blocked with 5% skimmed milk for 2 h at room temperature (RT) and then incubated with the primary antibodies at 4°C overnight. After three washes with TBST, the membranes were incubated with secondary antibodies for 2 h at RT. Following three additional TBST washes, immunoreactive bands were detected using an enhanced chemiluminescence (ECL) reagent (#BL520B; Biosharp) and visualized with a Fusion FX7 chemiluminescence imaging system (VILBER).

### 2.8. Immunofluorescence Assay (IFA) and Confocal Microscopy

The monolayer cells were washed with ice‐cold PBS, fixed in 4% paraformaldehyde (#BL539A; Biosharp) for 10 min, and permeabilized with 0.1% Triton X‐100 (#1139ML100; BioFroxx) for 15 min. After blocking with 5% BSA (#A8020; Solarbio) for 1 h, samples were incubated with primary and secondary antibodies. Nuclei were counterstained with DAPI (#C1002; Beyotime). Images were captured using a confocal microscope (CLSM610; SUNNY). Colocalization was quantified utilizing the JaCoP plugin within ImageJ software [35]. At least 10 cells in each of three independent experiments were randomly analyzed. Pearson’s colocalization coefficient (PCC) >0.5 indicates a correlation in intensity distribution between channels [36, 37]. Representative fluorescent images are displayed.

### 2.9. PRRSV Titration Assay

The infected cells were subjected to three freeze–thaw cycles and then centrifuged for the removal of cellular debris. The intracellular or extracellular infectious virions were titrated by determining 50% tissue culture infected dose (TCID_50_) in Marc145 cells. In brief, the cells seeded in 96‐well plates were inoculated with 10‐fold serial dilutions of PRRSV at 37°C for 1 h. After removing the uninternalized viruses with PBS, the cells were maintained in DMEM supplemented with 2% FBS for 3–5 days. The development of cytopathic effect was monitored daily, and the TCID_50_ was determined according to the Reed–Muench method.

### 2.10. Attachment and Entry Assay

PRRSV attachment and entry were assessed in cells transfected with siRNAs or pretreated with pharmacological inhibitors (sunitinib/ACA). For attachment, the cells were adsorbed with PRRSV strain BJ‐4 at a multiplicity of infection (MOI) of 5 at 4°C for 1 h and washed three times with ice‐cold PBS. The cells were harvested and the cell‐bound viral RNA levels were analyzed by RT‐qPCR, and the 200 μL cell lysates per well were collected to determine the cell‐associated infectious particles using TCID_50_ assay. For entry, following viral adsorption, the cells were shifted to 37°C for 0.5, 1, or 2 h, extensively washed with trypsin–PBS to remove the uninternalized viral particles, and the internalized viruses were similarly quantified by RT‐qPCR or TCID_50_ assay. Alternatively, PRRSV entry was evaluated by examining the colocalization between viral N protein and EEA1.

### 2.11. Replication, Assembly, and Release Assays

Marc145 cells were infected with BJ‐4 (MOI = 5) and incubated at 37°C for 1 h, after which the viral inoculum was substituted with medium containing 0.75 μM sunitinib. Viral replication was assessed using IFA by staining for the double‐stranded RNA (dsRNA) at 6, 8, and 10 h post‐infection (hpi). The efficiency of PRRSV assembly was indicated as the relative intracellular virus infectivity and determined at 10 hpi by comparing the intracellular virus infectivity (TCID_50_/mL) with the intracellular virus genomic equivalents. The efficiency of PRRSV release was quantified as the ratio of extracellular virus infectivity to the total virus infectivity (intracellular plus extracellular).

### 2.12. Co‐Immunoprecipitation (Co‐IP)

The Marc145 or iPAM cells were infected with PRRSV for 24 h, or the HEK‐293T cells were co‐expressed with Myc‐AP2M1 and Flag–GP5, or Flag–GP5 alone for 36 h. After washing with ice‐cold PBS, the cells were lysed in WB/IP lysis buffer (#P0013; Beyotime) for 30 min, then centrifuged at 12,000 × *g* for 10 min. The interaction between AP2M1 and GP5 or CD163 was assessed in accordance with the manufacturer’s protocol for BeyoMag Protein G beads (#P2105; Beyotime). Specifically, the supernatant was incubated with either mouse anti‐Flag MAb, rabbit anti‐Myc pAbs, or mouse anti‐CD163 MAb at RT for 2 h. Subsequently, 20 µL of immunomagnetic beads were added to the mixtures and co‐incubated at RT for an additional 2 h. The resulting immunocomplexes were isolated using a magnetic rack. The samples were ultimately analyzed via WB with the designated antibodies.

### 2.13. Statistical Analysis

In each experiment, three replicates were included, and each experiment was conducted at least three times independently. The experimental data are shown as group means with standard errors (SEM). Statistical analyses were conducted using GraphPad Prism software (Version 8.0) with unpaired two‐tailed Student’s *t*‐tests for two‐group comparisons and one‐way ANOVA with Dunnett’s test for multiple group comparisons. Statistical significance is denoted by asterisks ( ^∗^ *p* < 0.05;  ^∗∗^ *p* < 0.01;  ^∗∗∗^ *p* < 0.001;  ^∗∗∗∗^ *p* < 0.0001; ns, *p*  > 0.05, not significant).

## 3. Results

### 3.1. Certain NAKs Are Required for PRRSV Infection

We first evaluated the cytotoxicity and knockdown efficiency of siRNAs targeting members of the NAK family. As illustrated in Figure 1A, the tested siRNAs exhibited no significant cytotoxicity toward Marc145 cells (cell viability >90%) at 72 h post‐transfection (hpt). RT‐qPCR analysis verified that these siRNA duplexes effectively diminished their mRNA expression levels (Figure 1B). To explore the role of NAKs in PRRSV infection, the loss‐of‐function experiments were conducted utilizing RNA interference. Marc145 cells were transfected with the designated siRNAs or a negative control (siNC) for 24 h, followed by infection with PRRSV at an MOI of 0.1 for 24 h. For assessment of PRRSV infection, the cells were harvested and analyzed for intracellular viral RNA abundance via RT‐qPCR, intracellular viral N protein expression by WB, and extracellular viral titers using the TCID_50_ assay. The results indicated that knockdown of AAK1, GAK, and BMP2K, but not STK16, significantly inhibited PRRSV infection (Figure 1C–E). Specifically, knockdown of AAK1, GAK, and BMP2K resulted in marked reductions in viral RNA levels (49.7%, 47.4%, and 86.0%), viral N protein expression, and viral titers (1.0, 1.6, and 1.5 log_10_ TCID_50_/mL). Consistent with these findings, IFA further confirmed that deletion of NAKs reduced viral infection rate from 37.8% to 10.9%, 16.4%, and 12.6%, respectively (Figure 1F). Together, these data suggest that the NAK members AAK1, GAK, and BMP2K are required for PRRSV infection. In the following sections, the term NAKs specifically denotes AAK1, GAK, and BMP2K unless otherwise noted.

Figure 1Certain NAKs are required for PRRSV infection. (A) The Marc145 cells grown in 96‐well plates were transfected with the siRNA‐targeting individual NAKs (AAK1, GAK, BMP2K, or STK16) or a siNC for 72 h, and the cytotoxicity was evaluated using the CCK‐8 assay. The cell viabilities in siNC‐transfected cells were set to 100%. (B) The Marc145 cells grown in 24‐well plates were transfected with the indicated siRNAs for 24 h, and the silencing efficiency of the targeted kinases was evaluated by RT‐qPCR. (C–F) The siRNA‐transfected Marc145 cells were inoculated with PRRSV at an MOI of 0.1 for 24 h. (C) The intracellular PRRSV RNA abundance was determined by RT‐qPCR. (D) The PRRSV N protein expression was detected by WB, with β‐actin serving as a loading control. The band intensities of viral N protein were quantified and normalized to β‐actin. (E) The progeny PRRSV titers in culture supernatant were measured by assessing TCID_50_. (F) The PRRSV infectivity was visualized by IFA and calculated as the percentage of viral N protein‐positive cells (green) relative to total cells (nuclei stained with DAPI, blue). At least three images were analyzed from each of the three independent experiments. Representative images are shown. Scale bars, 50 μm. Data represent means ± SEM from three independent experiments. Statistical analysis was carried out using the one‐way ANOVA.  ^∗^ *p* < 0.05;  ^∗∗^ *p* < 0.01;  ^∗∗∗^ *p* < 0.001;  ^∗∗∗∗^ *p* < 0.0001; ns, not significant, *p* > 0.05.

**Figure:**
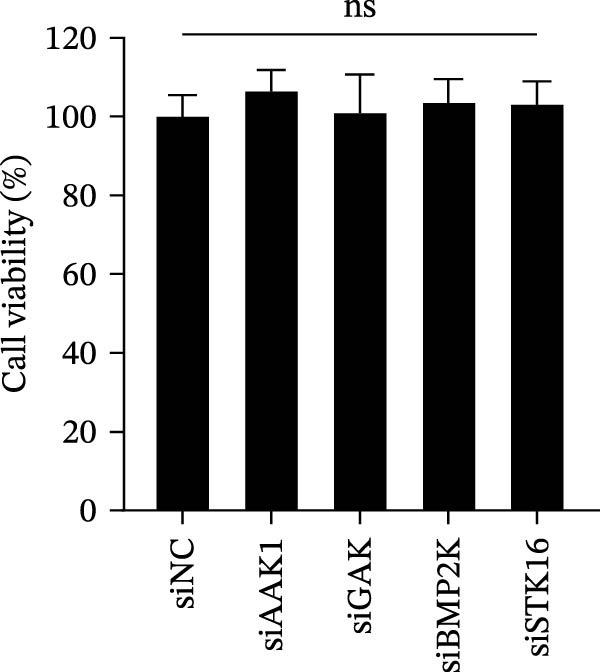
(A)

**Figure:**
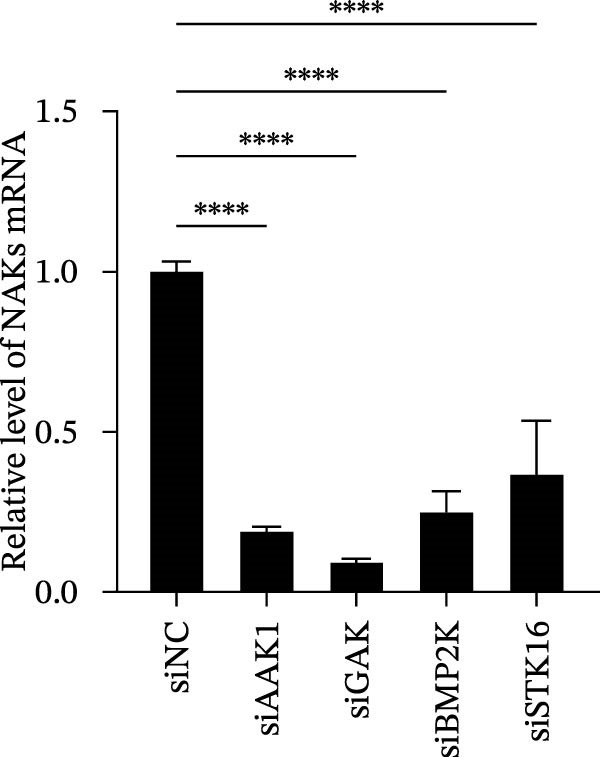
(B)

**Figure:**
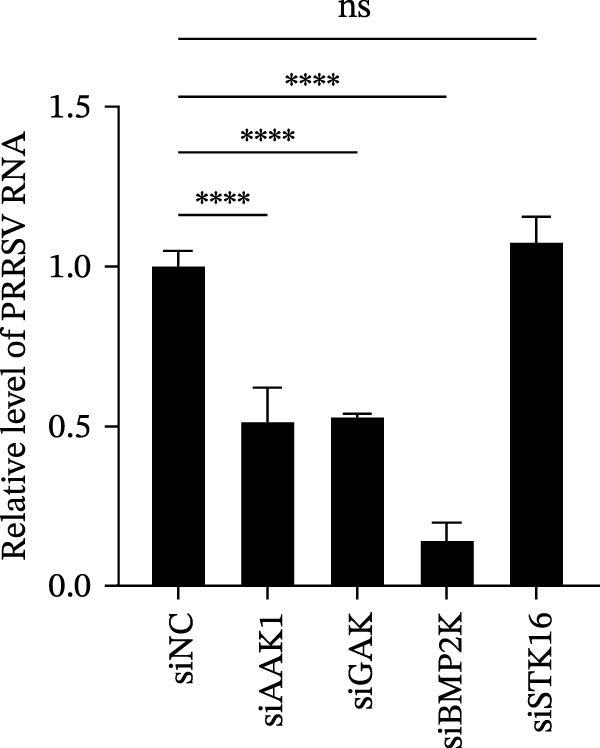
(C)

**Figure:**
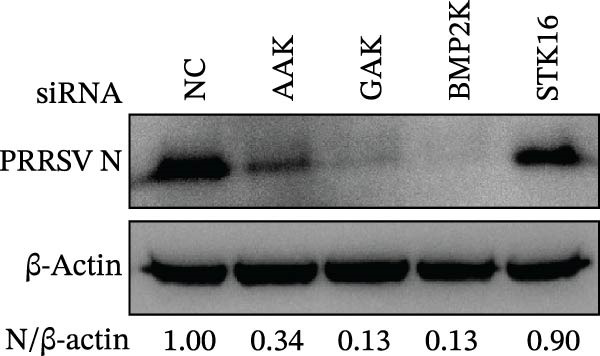
(D)

**Figure:**
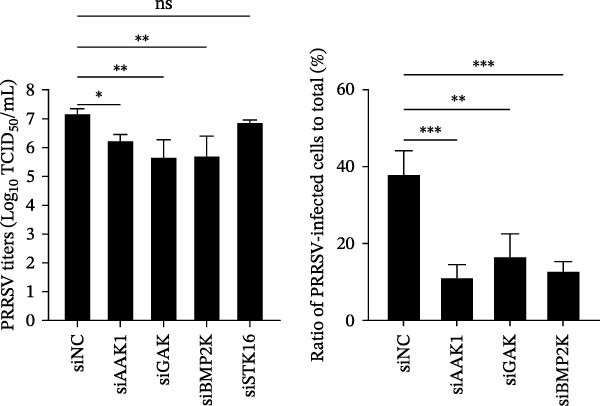
(E)

**Figure:**
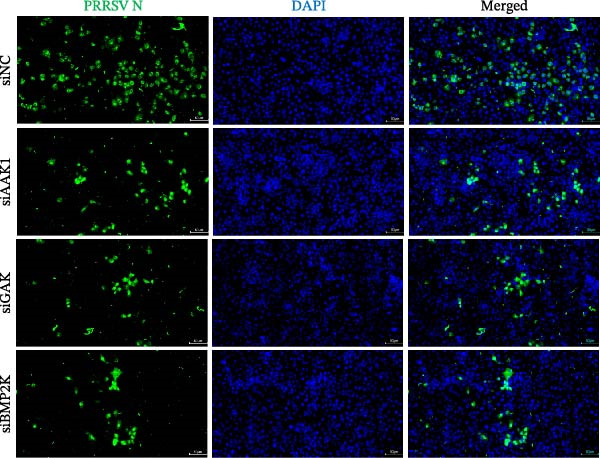
(F)

### 3.2. Pharmacological Inhibitors Targeting NAKs Suppress PRRSV Infection

To investigate whether the anti‐PRRSV effects observed in the genetic knockdown experiments could be recaptured pharmacologically, we examined the antiviral activity of two clinically approved NAK inhibitors, sunitinib and erlotinib. Previous research has established that the two inhibitors inhibit the kinase activity of AAK1, GAK, and BMP2K by binding to their conserved catalytic pockets [31, 33]. We first determined the noncytotoxic concentrations of sunitinib in two cell lines (≤0.75 μM in Marc145 cells, Figure 2A; ≤0. 5 μM in iPAM cells, Figure 2E). Marc145 and iPAM cells were then pretreated with increasing concentrations of sunitinib for 2 h, followed by infection with PRRSV (MOI = 0.1) for 24 h. The cells were subsequently harvested, and PRRSV infection was analyzed by WB, TCID_50_ assay, and/or IFA. Results showed that sunitinib markedly and dose‐dependently suppressed PRRSV infection in both cell lines, as evidenced by reduced viral N protein expression (Figure 2B,F), decreased viral titers (0.2, 1.4, and 1.6 log_10_ TCID_50_/mL in Marc145 cells, Figure 2C; 0.4, 0.4, and 0.9 log_10_ TCID_50_/mL in iPAM cells, Figure 2G), and diminished viral infectivity (12.7%, 55.1%, and 68.2% in Marc145 cells; Figure 2D). We next determined the noncytotoxic concentrations of erlotinib in two cell lines (≤1.5 μM in Marc145, Figure 2H; ≤0.25 μM in iPAM, Figure 2J). Similarly, erlotinib inhibited PRRSV infection in a dose‐dependent manner, as reflected by reduced viral titers of 0.1, 0.5, and 0.9 log_10_ TCID_50_/mL in Marc145 cells (Figure 2I) and 0.05, 0.1, and 0.6 log_10_ TCID_50_/mL in iPAM cells (Figure 2K). Taken together, these data further indicate that the involvement of NAKs in PRRSV infection is dependent on their kinase activity.

Figure 2Pharmacological inhibitors targeting NAKs suppress PRRSV infection. (A) The Marc145 cells were treated with increasing concentrations of sunitinib (0.1–1.00 μM) for 72 h, and the cytotoxicity was evaluated using the CCK‐8 assay. Cell viability was normalized to vehicle‐treated cells (0.02% DMSO), which was set to 100%. (B–D). The Marc145 cells were pretreated with sunitinib for 2 h, and then co‐inoculated with PRRSV (MOI = 0.1) and the inhibitor for 24 h. Cells treated with 0.02% DMSO served as the vehicle control. (B) The PRRSV N protein levels were determined by WB, with β‐actin used as a loading control. The band intensities of viral N protein were quantified and normalized to β‐actin. (C) The progeny PRRSV titers in culture supernatants were measured using the TCID_50_ assay. (D) The PRRSV infectivity was visualized by IFA and calculated as the percentage of viral N protein‐positive cells relative to total cells. (E) The iPAM cells were treated with the indicated concentrations of sunitinib for 72 h, followed by CCK‐8 analysis to determine cell viability. (F, G) The iPAM cells were pretreated with sunitinib for 2 h, and then co‐inoculated with PRRSV (MOI = 0.1) and the inhibitor for 24 h. (F) The PRRSV N protein levels were determined by WB. (G) The progeny PRRSV titers in culture supernatants were measured using the TCID_50_ assay. Marc145 (H) or iPAM (J) cells were treated with increasing concentrations of erlotinib for 72 h, and cell viability was measured using the CCK‐8 assay. The Marc145 (I) or iPAM (K) cells were pretreated with erlotinib for 2 h, and then co‐inoculated with PRRSV (MOI = 0.1) and the inhibitor for 24 h. The progeny PRRSV titers in culture supernatants were measured using the TCID_50_ assay. Data represent means ± SEM from three independent experiments. Statistical analysis was carried out using the one‐way ANOVA.  ^∗^ *p* < 0.05;  ^∗∗^ *p* < 0.01;  ^∗∗∗^ *p* < 0.001;  ^∗∗∗∗^ *p* < 0.0001; ns, not significant, *p* > 0.05.

**Figure:**
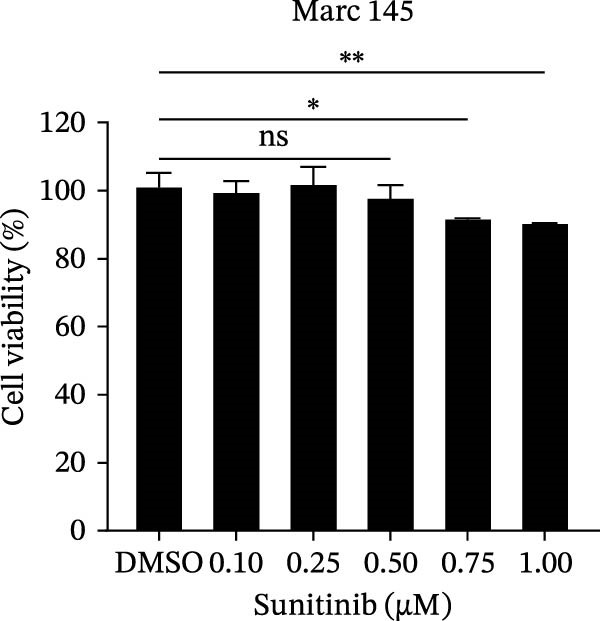
(A)

**Figure:**
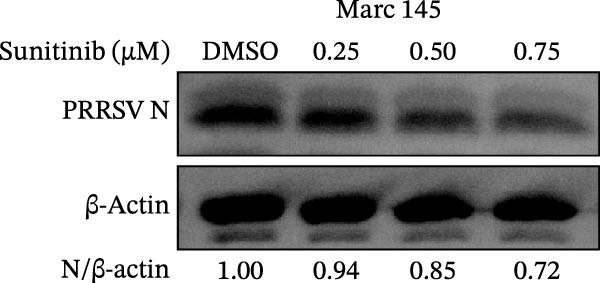
(B)

**Figure:**
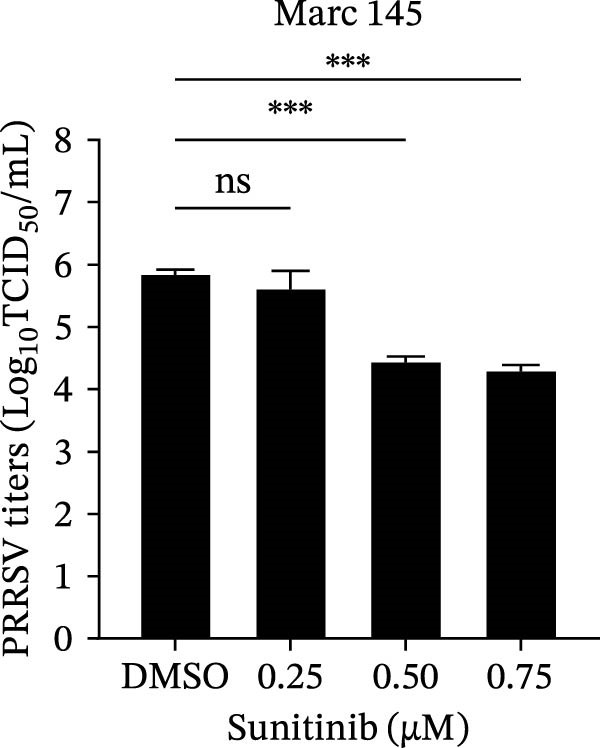
(C)

**Figure:**
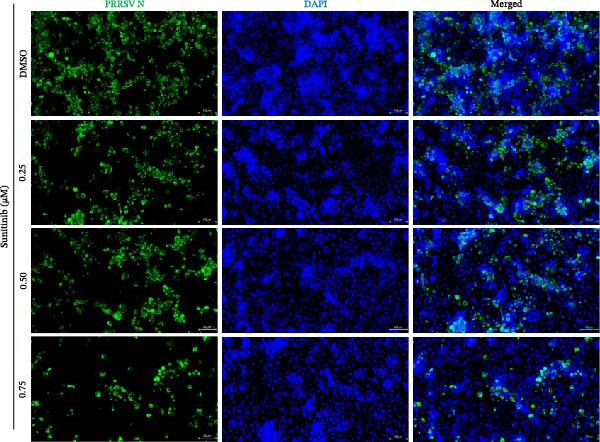
(D)

**Figure:**
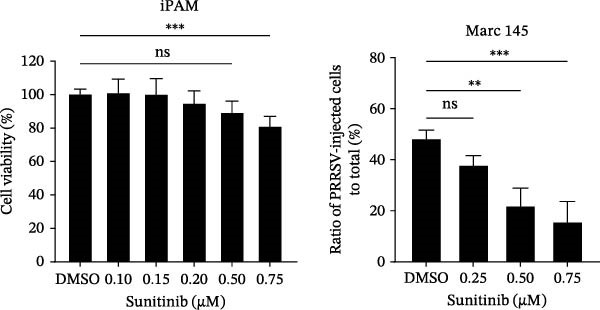
(E)

**Figure:**
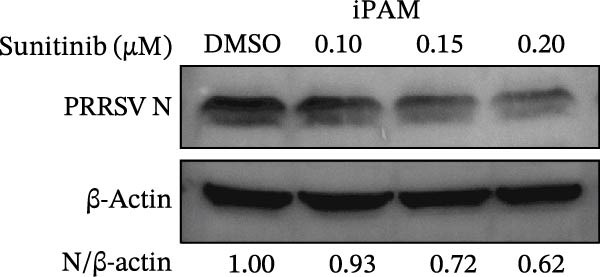
(F)

**Figure:**
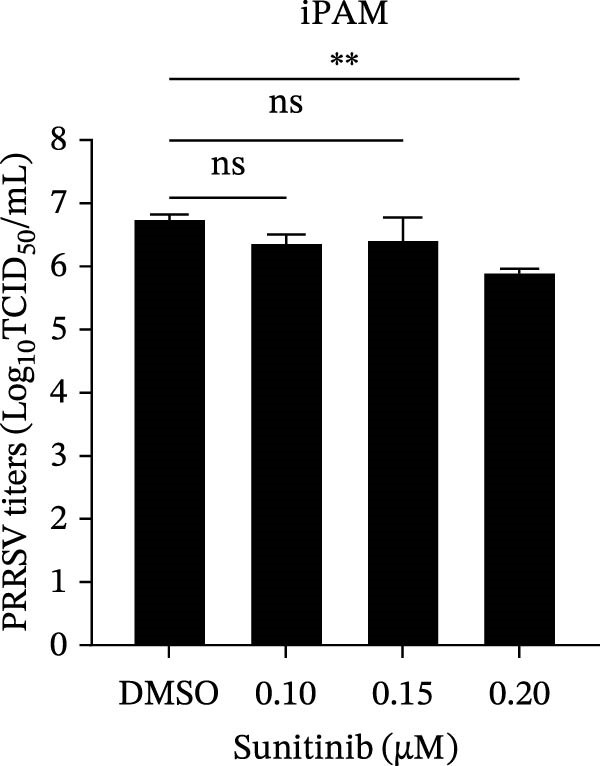
(G)

**Figure:**
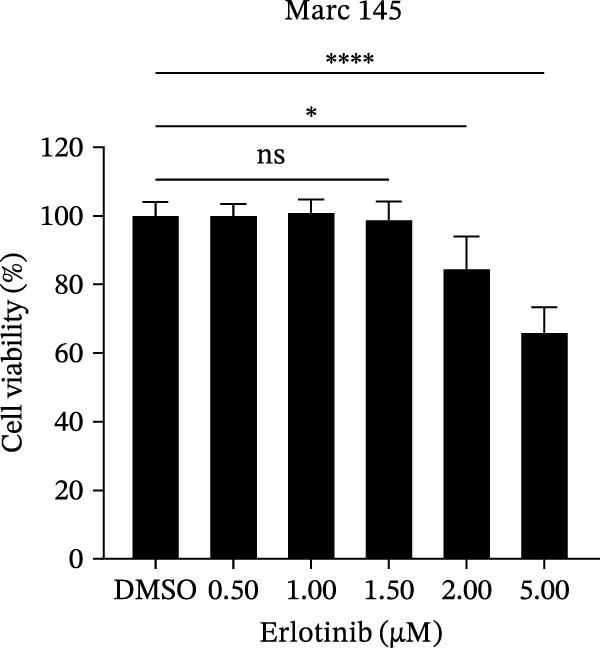
(H)

**Figure:**
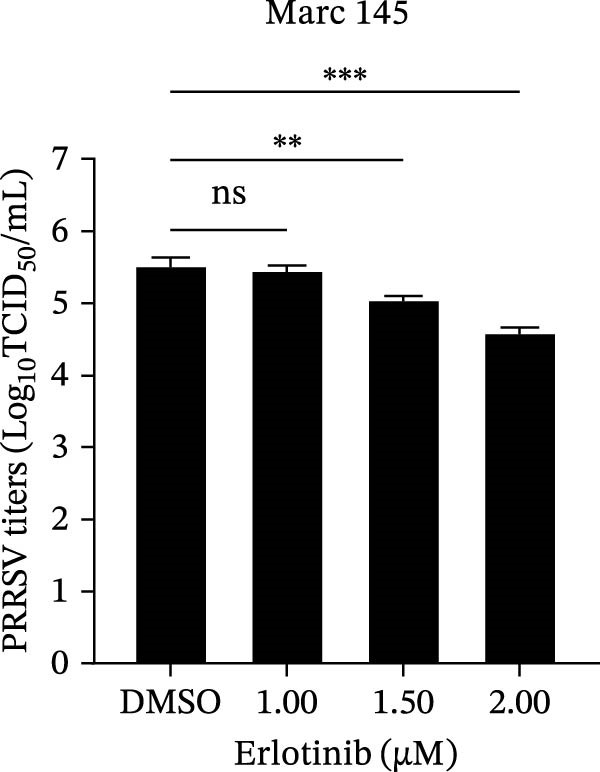
(I)

**Figure:**
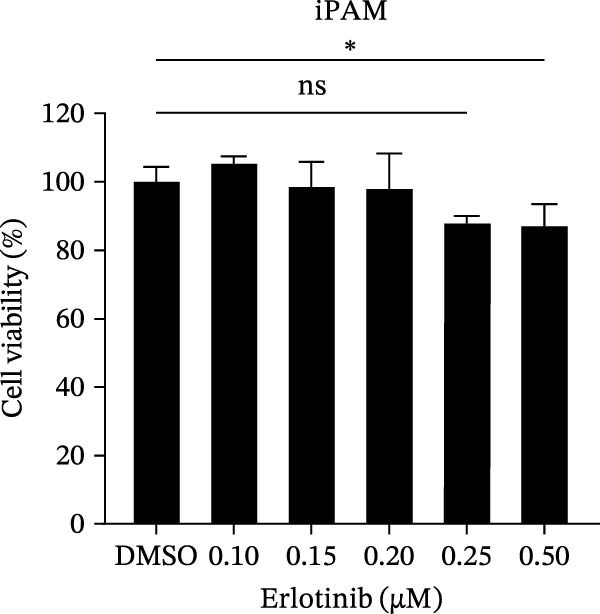
(J)

**Figure:**
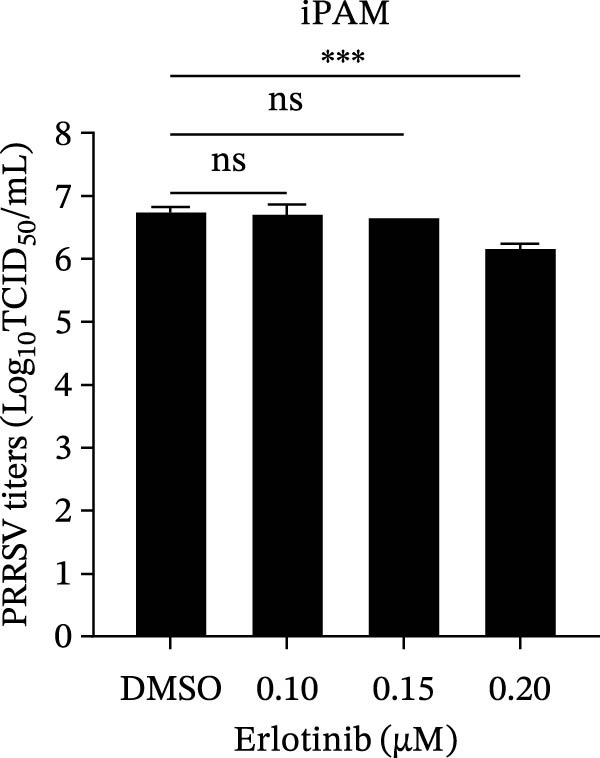
(K)

### 3.3. NAKs Play an Important Role in PRRSV Entry

To elucidate the specific stages of the PRRSV life cycle in which NAKs are involved, we initially explored their roles in viral attachment and entry. Marc145 cells with NAK knockdown were exposed to PRRSV at an MOI of 5 and maintained at 4°C for 1 h to permit virus adsorption without internalization. The amount of cell‐bound PRRSV was then assessed by RT‐qPCR and TCID_50_ assays. As shown in Figure 3A,B, knockdown of AAK1, GAK, or BMP2K did not significantly alter PRRSV RNA levels or viral titers, indicating that NAKs are dispensable for viral attachment. Next, we assessed the potential functional role of NAKs in PRRSV entry. After knocking down NAKs, the Marc145 cells were exposed to PRRSV at an MOI of 5 at 4°C for 1 h, followed by shifting to 37°C for 0.5, 1, or 2 h to allow virus internalization. RT‐qPCR analysis revealed that PRRSV RNA levels decreased by 34.5%, 22.3%, and 54.5% following knockdown of AAK1, GAK, and BMP2K, respectively (Figure 3C). Consistently, TCID_50_ assays demonstrated a significant reduction in viral titers in NAK‐depleted cells at indicated time points (> 0.3 log_10_ TCID_50_/mL; Figure 3D). In agreement with these findings, confocal microscopy exhibited that the PRRSV particles were partially retained at the cell periphery and showed reduced trafficking to the EEs (EEA1 as a marker of EEs) upon NAK deletion (Figure 3E, left panel). Quantitative colocalization analysis further revealed a decrease in PCC from 0.61 in control cells to < 0.5 in NAK‐silenced cells (Figure 3E, right panel).

Figure 3NAKs are involved in PRRSV entry. (A, B) The Marc145 cells were transfected with the indicated siRNAs for 24 h, infected with PRRSV (MOI = 5), and then incubated at 4°C for 1 h to allow viral binding without internalization. After washing with ice‐cold PBS, PRRSV attachment was evaluated by detecting the cell‐bound viral RNA abundance using RT‐qPCR (A) or the 200 μL cell lysates per well were collected to determine viral titers using TCID_50_ assay (B). (C–E) The siRNA‐transfected Marc145 cells were infected or not infected with PRRSV (MOI = 5) at 4°C for 1 h. After washing away the unbound virions, the cells were shifted to 37°C for 0.5, 1, or 2 h to allow virus internalization. (C) At 1 hpi, PRRSV entry was assessed by detecting the internalized viral RNA abundance by RT‐qPCR. (D) At 0.5, 1, or 2 hpi, the medium was discarded and 200 μL cell lysates per well were collected to determine the internalized viral titers using TCID_50_ assay. (E) The subcellular localization of viral N protein with EEA1 was analyzed by confocal microscopy (EEA1: green; N protein: red; nuclei: blue; scale bars: 10 μm). The colocalization was assessed by determining the PCC using the ImageJ software. At least 10 cells in each of three independent experiments were randomly analyzed. PCC >0.5 describes the correlation of the intensity distribution between channels. (F, G) The Marc145 cells were pretreated with sunitinib for 2 h, and then co‐incubated with PRRSV (MOI = 5) and the inhibitor at 4°C for 1 h. After washing with ice‐cold PBS, PRRSV attachment was evaluated by detecting the cell‐bound viral RNA abundance using RT‐qPCR (F) or viral titers using TCID_50_ assay (G). (H–J) The sunitinib‐pretreated Marc145 cells were infected or not infected with PRRSV (MOI = 5) at 4°C for 1 h, and then shifted to 37°C for 1 h to allow internalization. PRRSV entry was evaluated by measuring the internalized viral RNA abundance by RT‐qPCR (H), the viral titers by TCID_50_ assay (I), or the subcellular localization of viral N protein with EEA1 by confocal microscopy (EEA1: green; N protein: red; nuclei: blue; scale bars: 10 μm) (J). The colocalization was assessed by determining the PCC using the ImageJ software. Data represent means ± SEM from three independent experiments. Statistical analysis was carried out using the one‐way ANOVA or Student’s *t*‐test.  ^∗^ *p* < 0.05;  ^∗∗^ *p* < 0.01;  ^∗∗∗^ *p* < 0.001;  ^∗∗∗∗^ *p* < 0.0001; ns, not significant, *p* > 0.05.

**Figure:**
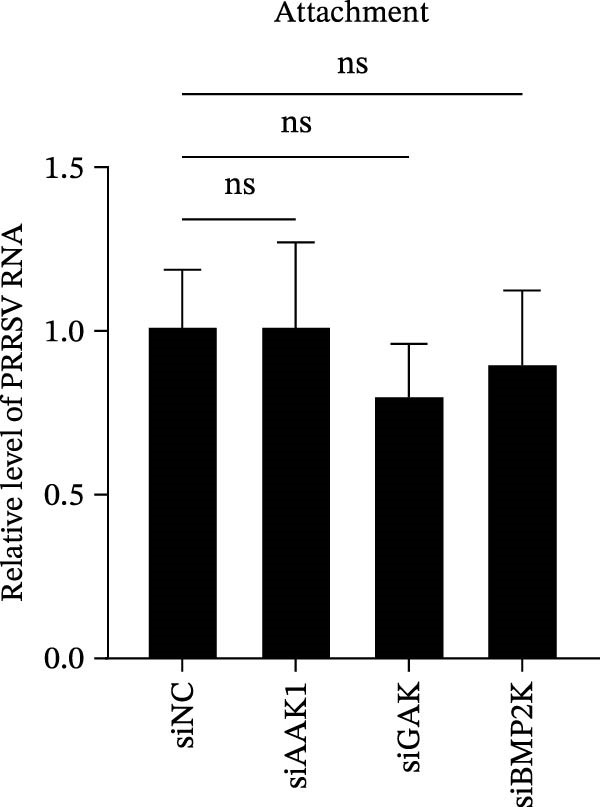
(A)

**Figure:**
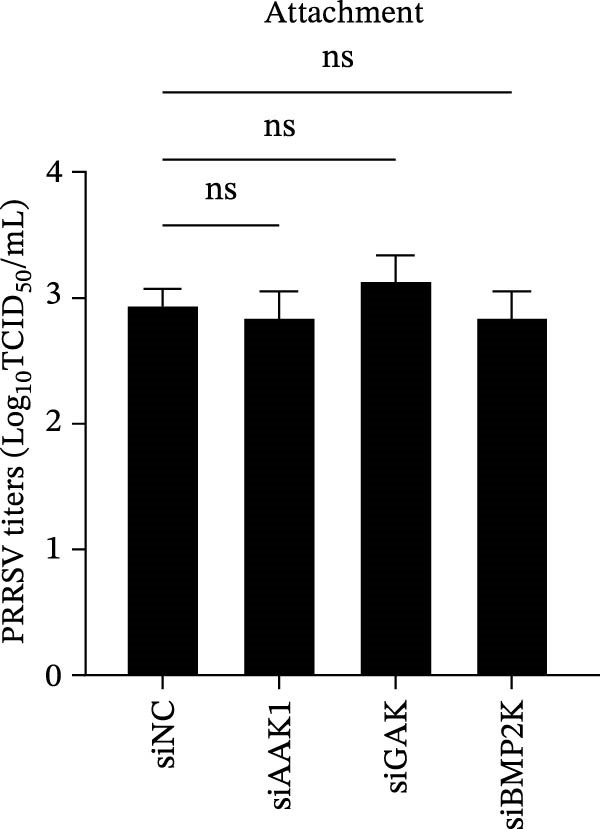
(B)

**Figure:**
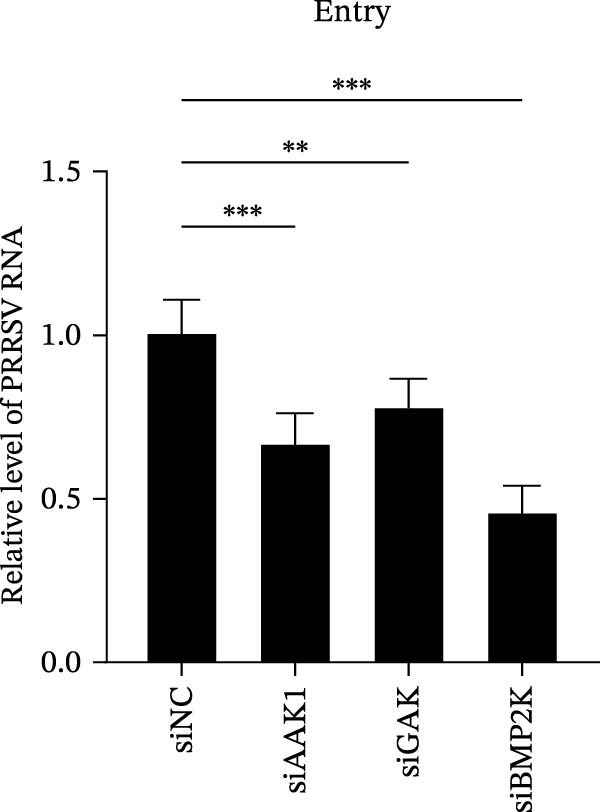
(C)

**Figure:**
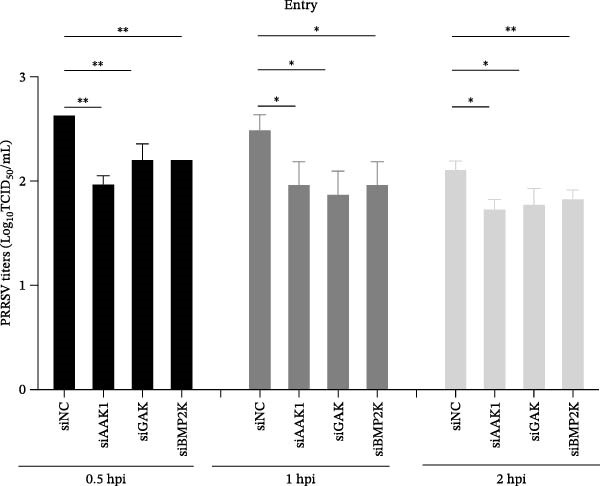
(D)

**Figure:**
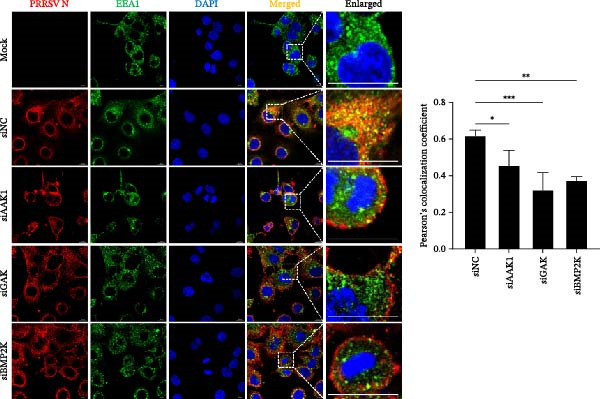
(E)

**Figure:**
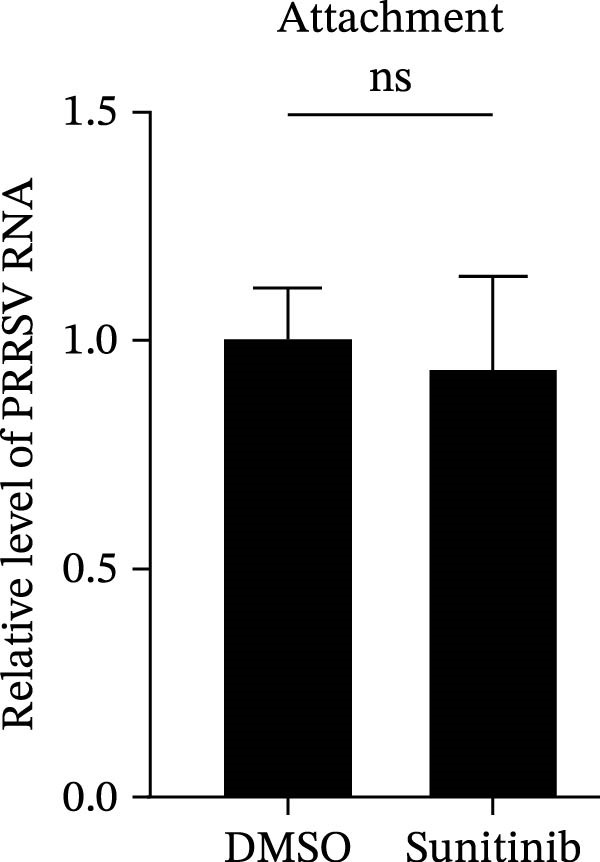
(F)

**Figure:**
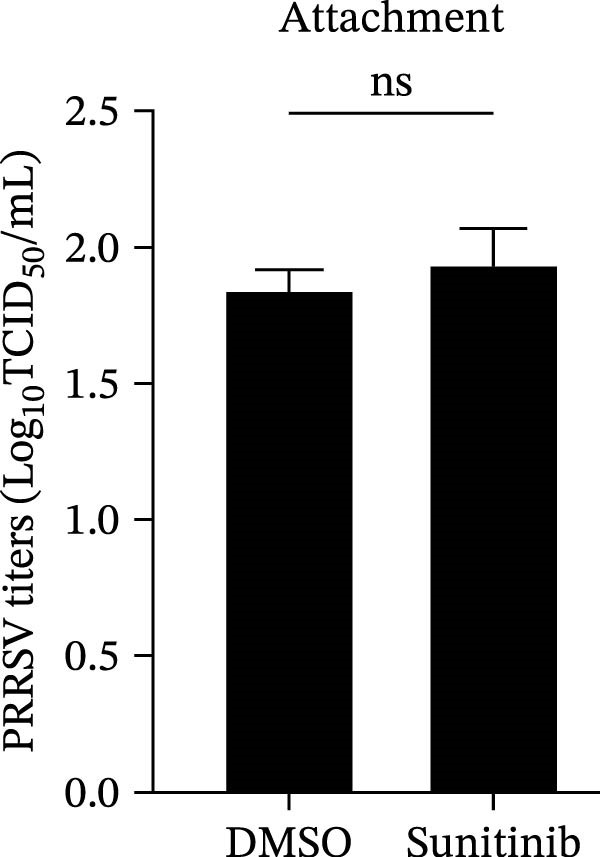
(G)

**Figure:**
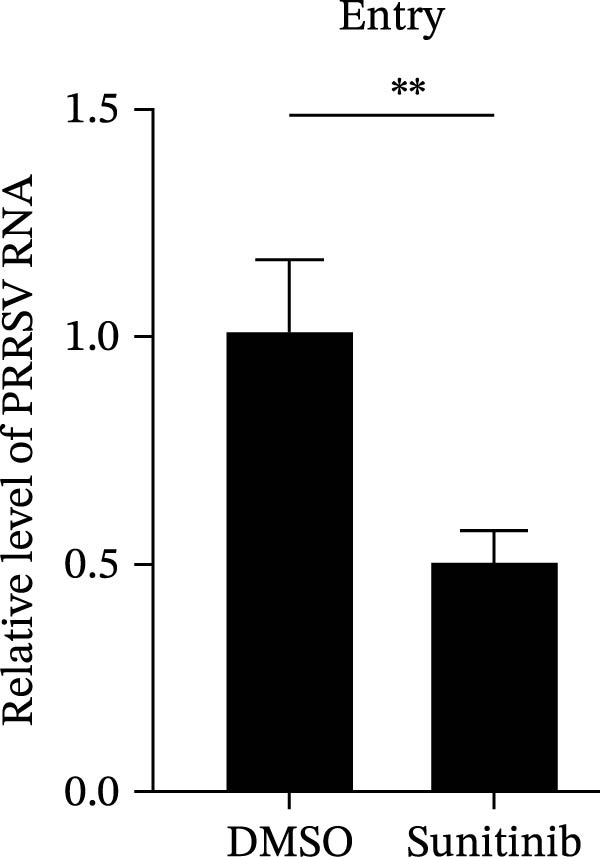
(H)

**Figure:**
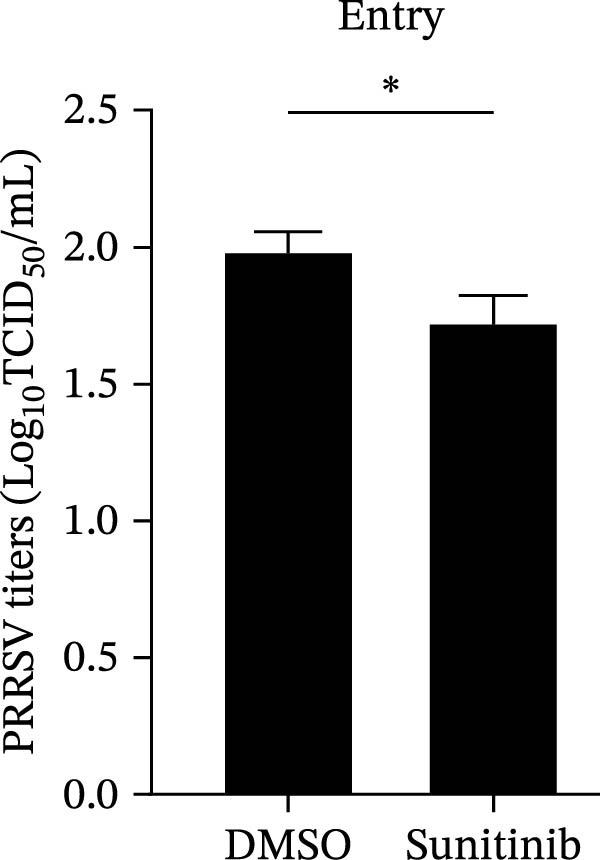
(I)

**Figure:**
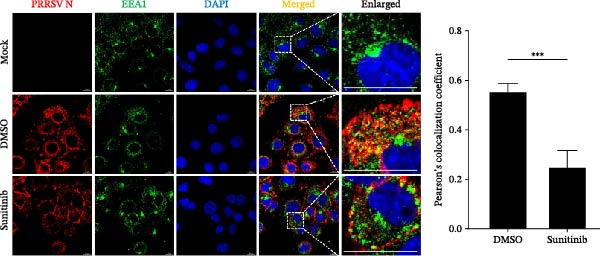
(J)

To further validate these findings, we conducted attachment and entry experiments using the NAK inhibitor sunitinib. The sunitinib‐pretreated Marc145 cells were incubated with PRRSV at 4°C for 1 h to assess virus attachment, or the cells were then transferred to 37°C for another 1 h to evaluate the subsequent entry. Consistent with the genetic knockdown results, sunitinib had no detectable effect on PRRSV attachment but notably obstructed the viral entry process, as evidenced by comparable PRRSV RNA levels and viral titers between DMSO‐ and sunitinib‐treated cells (Figure 3F,G). In contrast, sunitinib impaired PRRSV entry, leading to a reduction in internalized viral RNA levels (49.8%; Figure 3H) and viral titers (0.3 log_10_ TCID_50_/mL; Figure 3I). Confocal imaging further confirmed reduced colocalization of PRRSV N with EEA1‐positive EEs, with PCC decreasing from 0.55 to 0.25 (Figure 3J).

To explore whether NAKs also play roles in post‐entry stages of the PRRSV life cycle, we conducted drug‐addition experiments within the first viral replication cycle, which has been determined to be approximately 10 h [38, 39]. First, we evaluated the impact of sunitinib on viral RNA replication by monitoring the formation of dsRNA, a hallmark of positive‐strand RNA virus replication [40], at 6, 8, and 10 hpi. IFA analysis indicated that sunitinib did not significantly affect dsRNA synthesis at any examined time point, with comparable fluorescence intensities observed between the DMSO‐ and sunitinib‐treated groups (Figure 4A). Consistently, RT‐qPCR analysis demonstrated that there was no notable difference in the intracellular viral RNA accumulation between the two groups over time (Figure 4B). To further evaluate sunitinib’s effect on PRRSV assembly and release, the viral titers were determined in both intracellular and extracellular fractions at 10 hpi. Viral titration data showed that sunitinib treatment resulted in increased intracellular PRRSV titers (0.3 log_10_ TCID_50_/mL; Figure 4C) but reduced extracellular titers (0.6 log_10_ TCID_50_/mL; Figure 4D). Further analyses confirmed that sunitinib did not affect PRRSV assembly (Figure 4E) but slightly inhibited virus release (14.6%; Figure 4F). All together, these data demonstrate that NAKs primarily function at the viral entry stage during the PRRSV life cycle.

Figure 4NAKs are not involved in PRRSV replication and assembly, but have slight effects on viral release. (A, B) The Marc145 cells were infected with PRRSV (MOI = 5) at 37°C for 1 h. After washing, the inoculum was replaced with the fresh medium containing sunitinib, and the cells were further incubated at 37°C for the indicated times (6, 8, or 10 h). (A) PRRSV RNA replication was assessed using IFA with staining of the dsRNA at the indicated time points (dsRNA: green; nuclei: blue; scale bars: 50 μm). The relative fluorescence density was calculated by measuring the ratio of dsRNA‐positive cells (green) fluorescence intensities to total cells (blue), and the fluorescence intensity of the control group at 6 hpi was set for 1 for normalization. (B) The intracellular PRRSV RNA copy numbers were assessed by RT‐qPCR at the indicated time points. The intracellular (C) or extracellular (D) PRRSV titers were determined by TCID_50_ assay at 10 hpi. (E) PRRSV assembly efficiency was indicated by the relative intracellular specific infectivity and determined by comparing the intracellular infectious titers (TCID_50_/mL) to the intracellular viral genomic equivalents (GE). (F) PRRSV release efficiency was calculated as the ratio of intracellular and extracellular infectivity relative to the total viral infectivity. Data represent means ± SEM from three independent experiments. Statistical analysis was carried out using the Student’s *t*‐test.  ^∗^ *p* < 0.05;  ^∗∗∗^ *p* < 0.001;  ^∗∗∗∗^ *p* < 0.0001; ns, not significant, *p* > 0.05.

**Figure:**
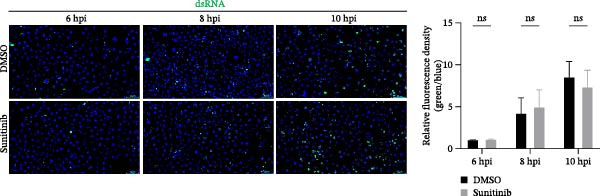
(A)

**Figure:**
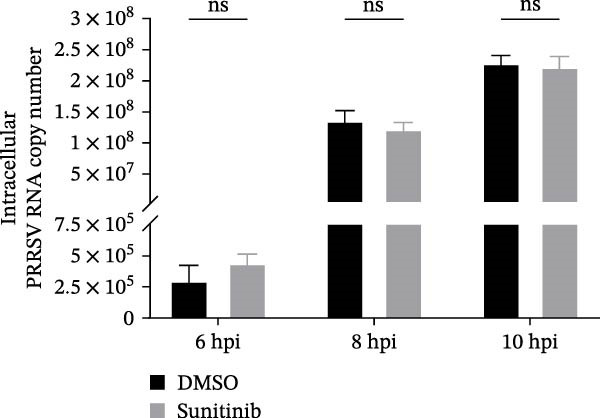
(B)

**Figure:**
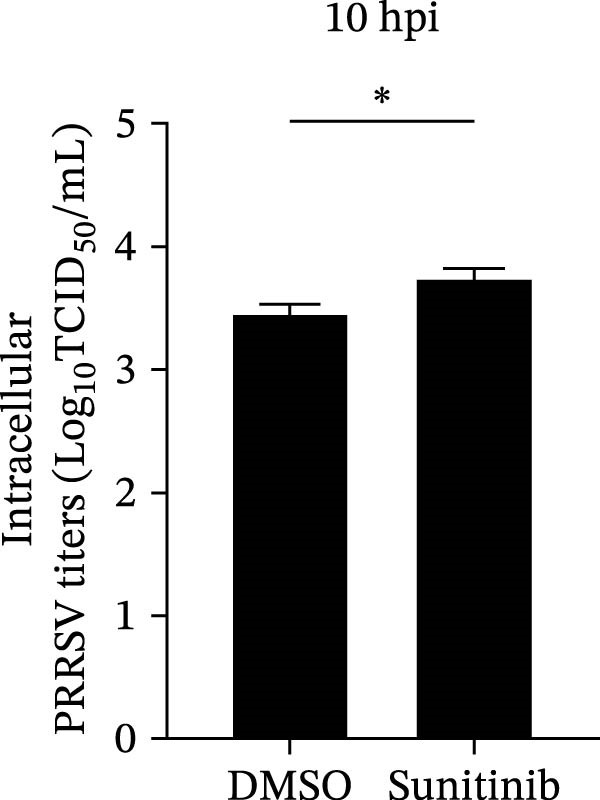
(C)

**Figure:**
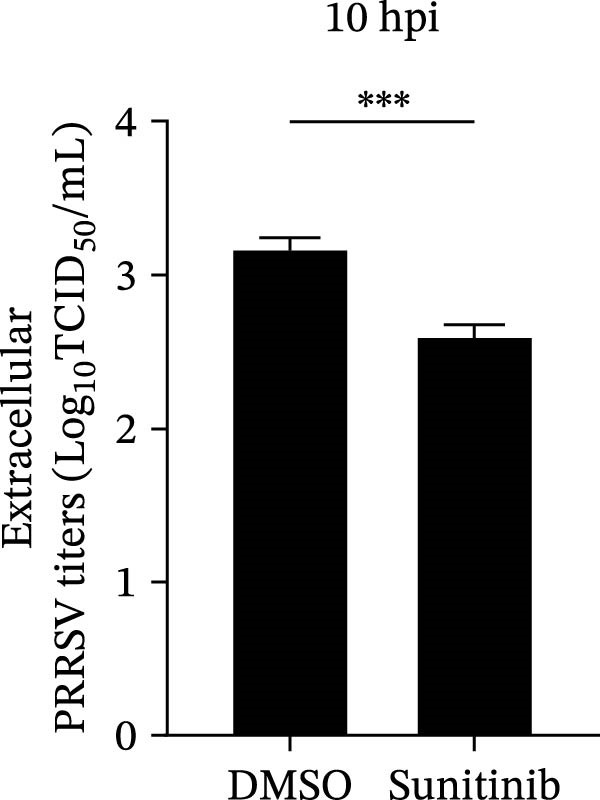
(D)

**Figure:**
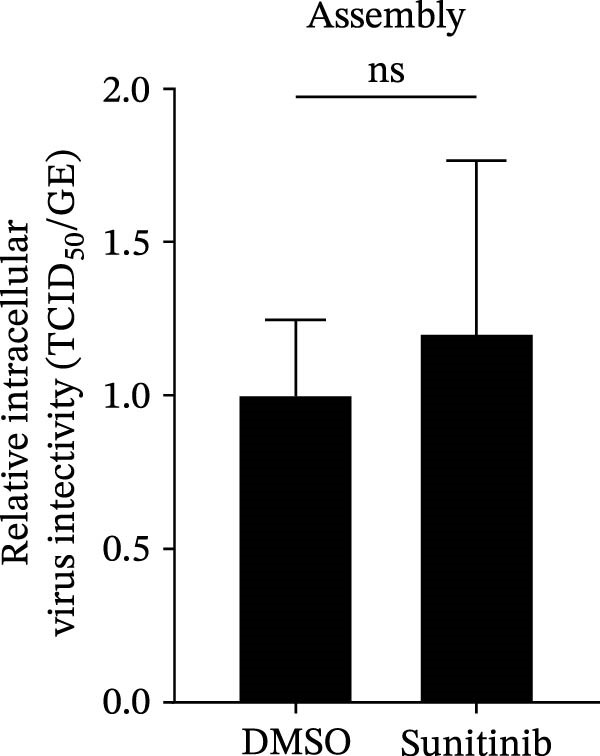
(E)

**Figure:**
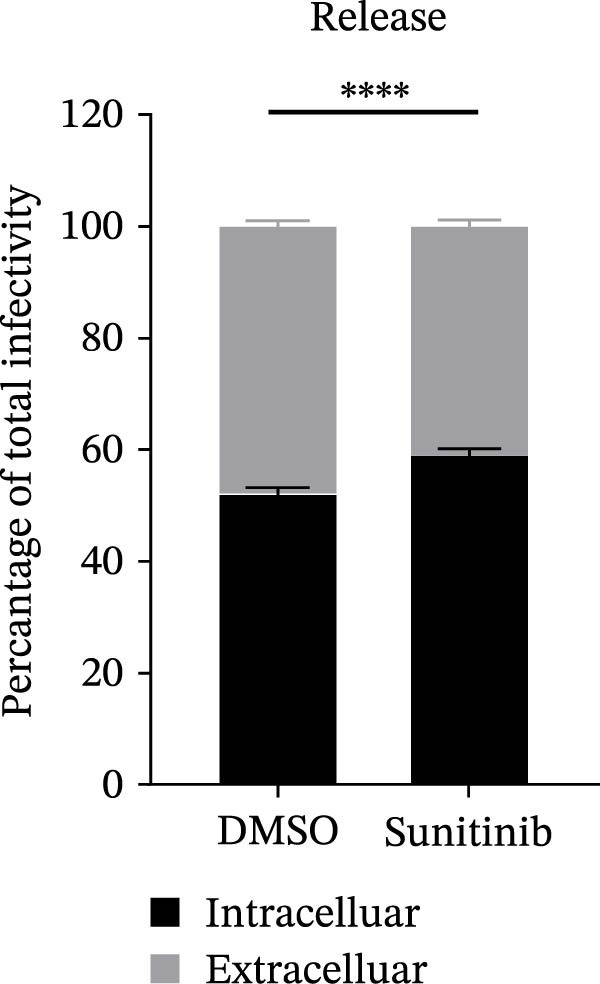
(F)

### 3.4. NAKs Promote PRRSV Entry Through AP2M1

Previous studies have identified that AP2M1, a core member of the adaptor protein complex family, is the major substrate protein of NAKs, and they synergistically regulate endocytosis of cargo proteins [21–23]. Here, we first examined whether NAKs regulate AP2M1 phosphorylation in Marc145 cells. RT‐qPCR and WB analyses revealed that knockdown of AAK1, GAK, or BMP2K did not alter the transcription or total protein levels of AP2M1 but reduced its phosphorylation at the T156 residue by 25.0%, 19.7%, and 32.3%, respectively (Figure 5A–C). Consistently, sunitinib treatment had no effect on AP2M1 mRNA or total protein levels at 2–4 h post‐treatment but decreased its phosphorylation by 17.4% and 20.3% (Figure 5D–F).

Figure 5NAKs regulate the phosphorylation of AP2M1 at the T156 residue. (A–C) The Marc145 cells were transfected with siRNAs targeting AAK1, GAK, or BMP2K for 24 h, and then collected for subsequent detection. (A) The mRNA levels of AP2M1 were determined by RT‐qPCR. (B) Total AP2M1 and the phosphorylated AP2M1 (pAP2M1 T156) protein levels were detected using WB. (C) Quantitative densitometric analysis of AP2M1 phosphorylation, expressed as the ratio of pAP2M1 (T156) to total AP2M1 protein. Values were normalized to the siNC‐transfected group, which was set to 1.0. (D–F) The Marc145 cells were pretreated with sunitinib (0.75 μM) for 2 or 4 h before harvest. (D) The mRNA levels of AP2M1 were determined by RT‐qPCR following sunitinib treatment. (E) Total AP2M1 and the phosphorylated AP2M1 (pAP2M1 T156) protein levels were detected using WB. (F) Quantitative densitometric analysis of AP2M1 phosphorylation. Values were normalized to the 2 h DMSO‐treated control group, which was set to 1.0. Data represent means ± SEM from three independent experiments. Statistical analysis was carried out using the one‐way ANOVA or Student’s *t*‐test.  ^∗∗^ *p* < 0.01;  ^∗∗∗^ *p* < 0.001;  ^∗∗∗∗^ *p* < 0.0001; ns, not significant, *p* > 0.05.

**Figure:**
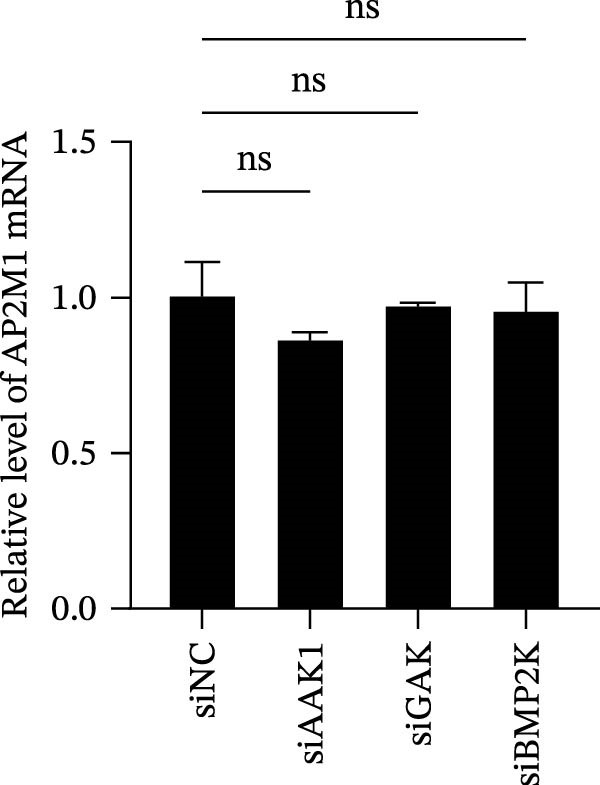
(A)

**Figure:**
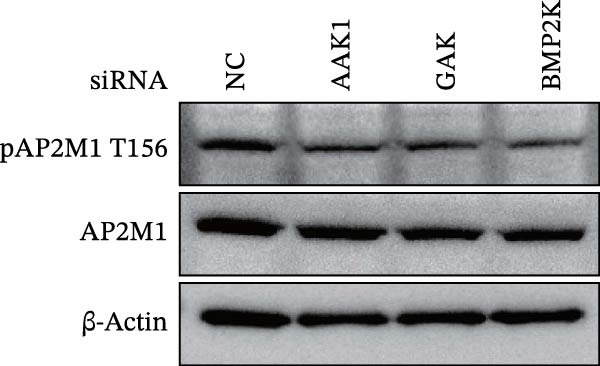
(B)

**Figure:**
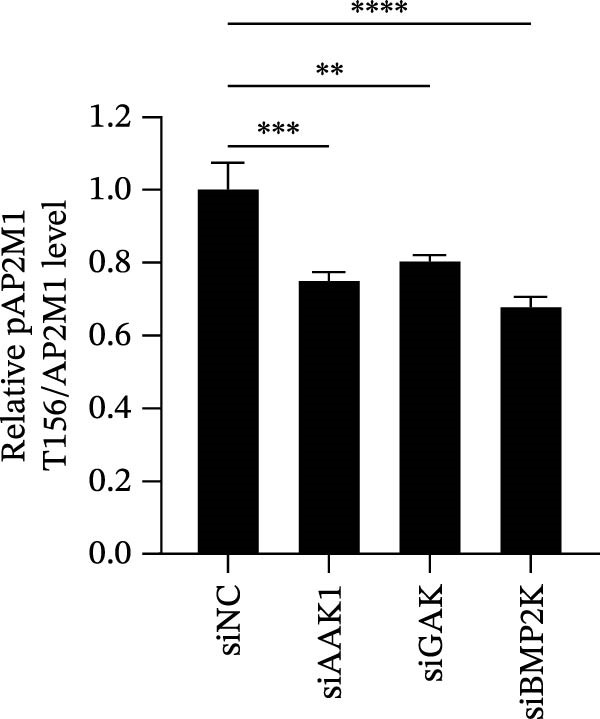
(C)

**Figure:**
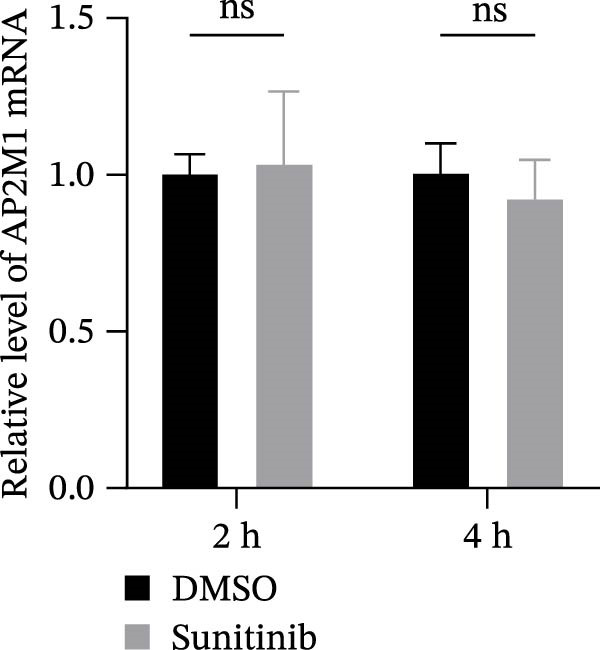
(D)

**Figure:**
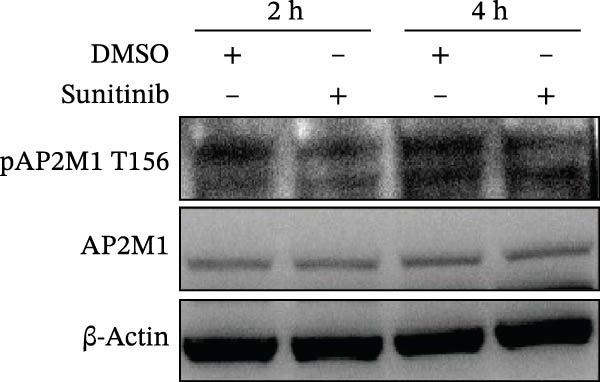
(E)

**Figure:**
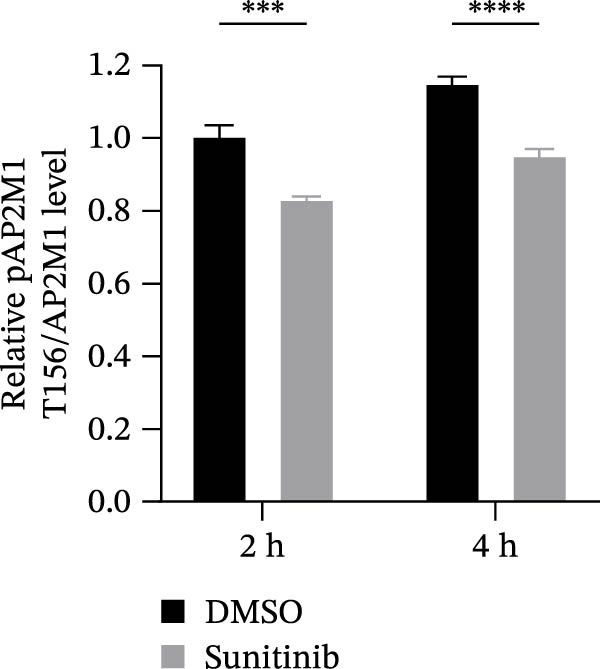
(F)

Given the critical role of the NAKs–AP2M1 axis, we further investigated whether AP2M1 contributed to PRRSV infection using knockdown, overexpression, and rescue experiments. Results showed that knockdown of AP2M1 caused a marked decline in AP2M1 mRNA and protein levels (Figure 6A,B), along with decreased PRRSV RNA levels (66.2%; Figure 6C), N protein expression (Figure 6D), and viral titers (1.6 log_10_ TCID_50_/mL; Figure 6E). Conversely, overexpression of AP2M1 markedly enhanced PRRSV infection, with increasing viral RNA abundance (3.1‐fold; Figure 6F), N protein expression (Figure 6G), and viral titers (0.9 log_10_ TCID_50_/mL; Figure 6H). Furthermore, reintroduction of a siAP2M1‐resistant construct in the AP2M1‐silenced cells restored viral N protein expression and virus production to near control levels (Figure 6I, J). Additionally, knocking down AP2M1 in iPAM cells exhibited similar inhibitory effects, as evidenced by reduced PRRSV RNA levels (58.3%; Figure 6K), diminished N protein expression (Figure 6L), and lower virus titers (1.6 log_10_ TCID_50_/mL; Figure 6M).

Figure 6AP2M1 is essential for PRRSV entry during infection. (A–E) The Marc145 cells were transfected with siAP2M1 or siNC for 24 h, and then uninfected or infected with PRRSV (MOI = 0.1) for 24 h. The knockdown efficiency of siAP2M1 was assessed by measuring AP2M1 mRNA levels using RT‐qPCR (A) and protein levels by WB (B). PRRSV infection was evaluated by detecting the intracellular viral RNA abundance using RT‐qPCR (C), viral N protein expression using WB (D), and extracellular viral titers using TCID_50_ assay (E). The band intensities of AP2M1 or N protein were quantified and normalized to β‐actin. (F–H) The Marc145 cells were transfected with Myc‐AP2M1 or empty vector (Myc) for 24 h, and then uninfected or infected with PRRSV (MOI = 0.1) for 24 h. PRRSV infection was evaluated by detecting the intracellular viral RNA abundance using RT‐qPCR (F), viral N protein expression using WB (G), and extracellular viral titers using TCID_50_ assay (H). The AP2M1‐silenced Marc145 cells were transfected with either Myc‐AP2M1 or Myc, followed by infection with PRRSV (MOI = 0.1) for 24 h, and the viral infection was assessed by WB (I) and TCID_50_ assay (J). The band intensities of N protein or AP2M1 were quantified and normalized to β‐actin. The AP2M1‐silenced iPAM cells were infected with PRRSV (MOI = 0.1) for 24 h, and the PRRSV infection was evaluated by detecting the intracellular viral RNA abundance using RT‐qPCR (K), viral N protein expression using WB (L), and extracellular viral titers using TCID_50_ assay (M). The AP2M1‐silenced Marc145 cells were infected with PRRSV (MOI = 5) at 4°C for 1 h. PRRSV attachment was evaluated by detecting the cell‐bound viral RNA abundance using RT‐qPCR (N) or viral titers using TCID_50_ assay (O). (P–R) The AP2M1‐silenced Marc145 cells were infected or not infected with PRRSV (MOI = 5) at 4°C for 1 h. After washing away the unbound viral particles, cells were shifted to 37°C for 1 h to allow internalization. PRRSV entry was assessed by detecting the internalized viral RNA abundance using RT‐qPCR (P), viral titers using TCID_50_ assay (Q), or the subcellular localization of viral N protein with EEA1 using confocal microscopy (EEA1: green; N protein: red; nuclei: blue; scale bars: 10 μm) (R). The colocalization was assessed by determining the PCC using the ImageJ software. Data represent means ± SEM from three independent experiments. Statistical analysis was carried out using the Student’s *t*‐test.  ^∗^ *p* < 0.05;  ^∗∗^ *p* < 0.01;  ^∗∗∗^ *p* < 0.001;  ^∗∗∗∗^ *p* < 0.0001; ns, not significant, *p* > 0.05.

**Figure:**
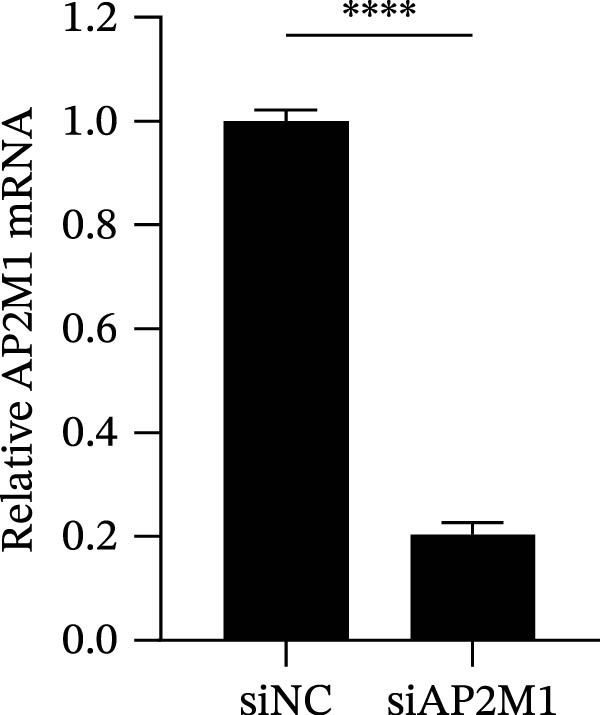
(A)

**Figure:**
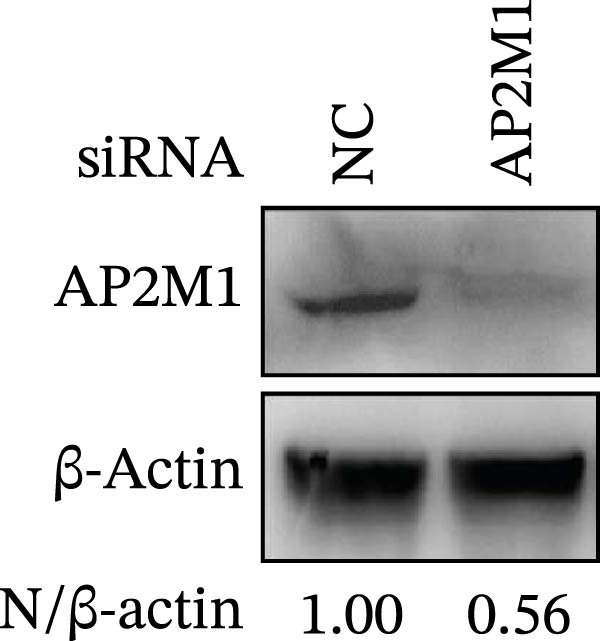
(B)

**Figure:**
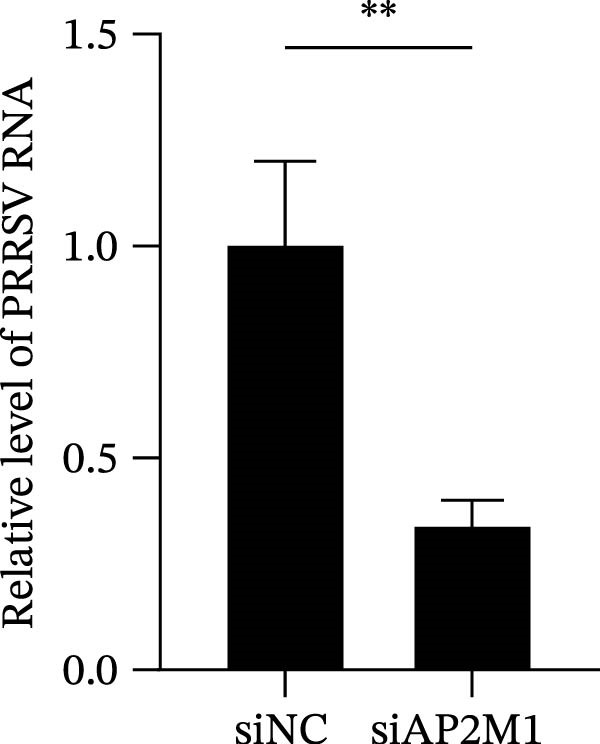
(C)

**Figure:**
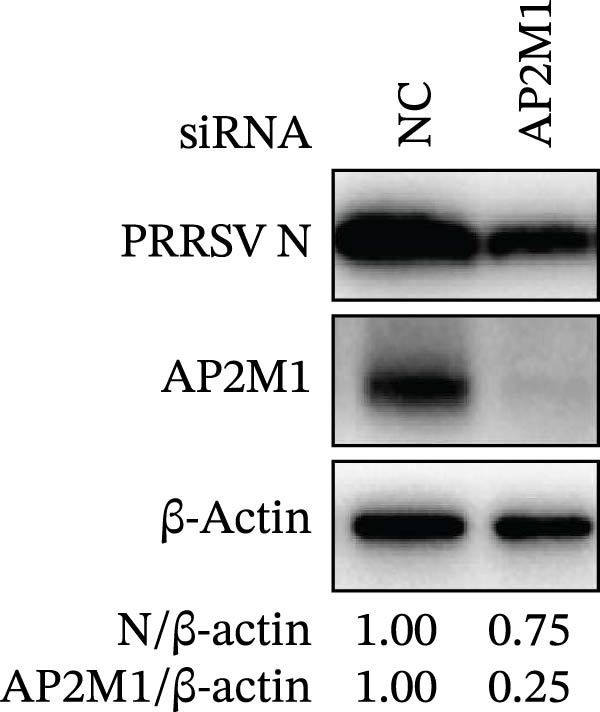
(D)

**Figure:**
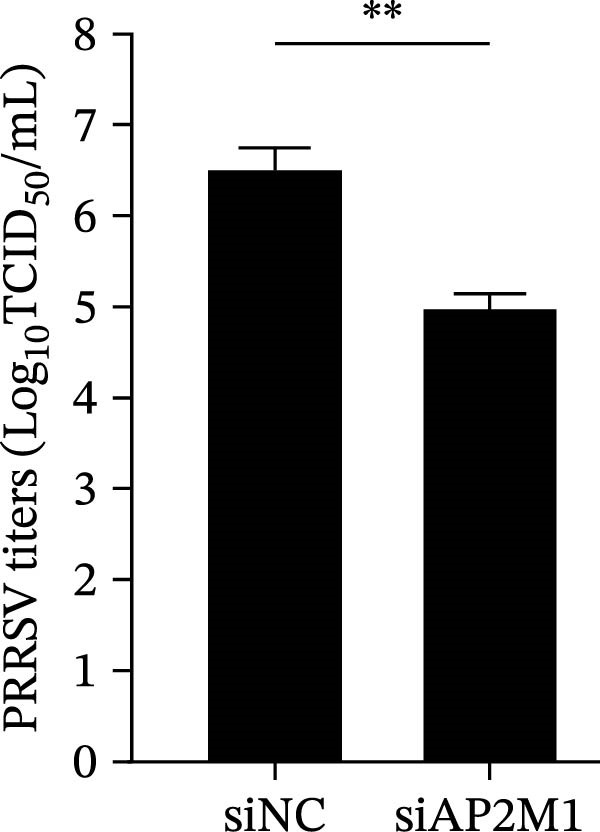
(E)

**Figure:**
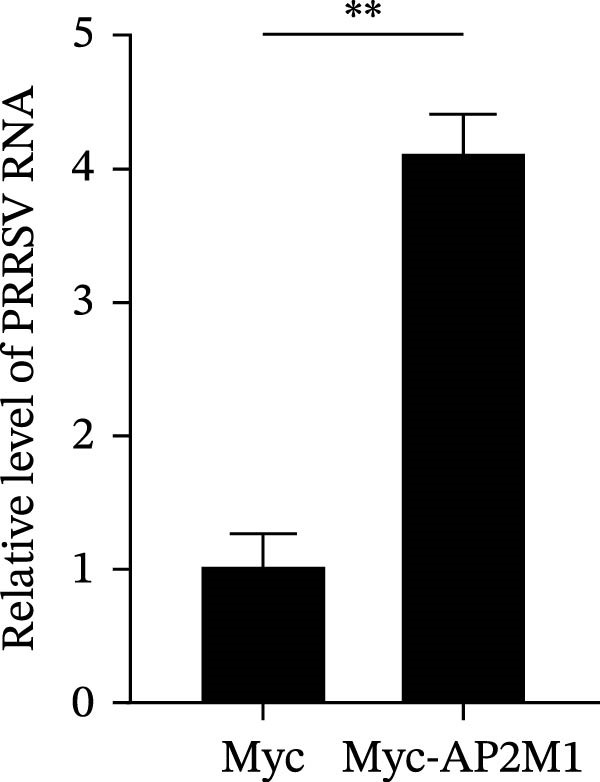
(F)

**Figure:**
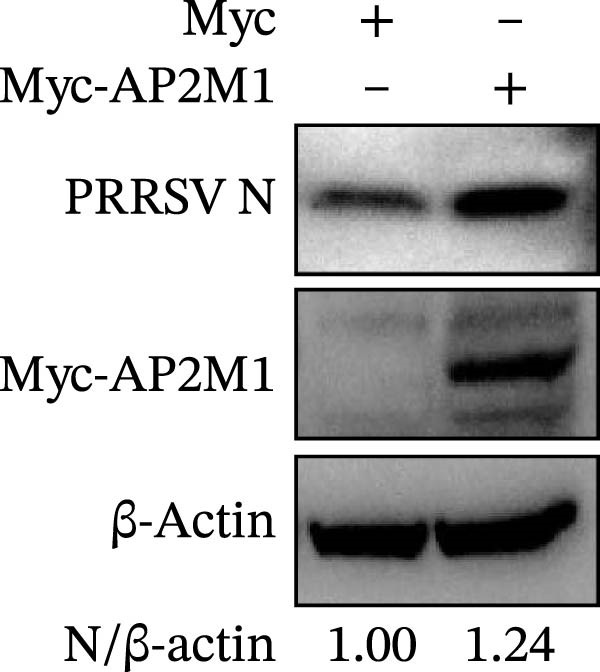
(G)

**Figure:**
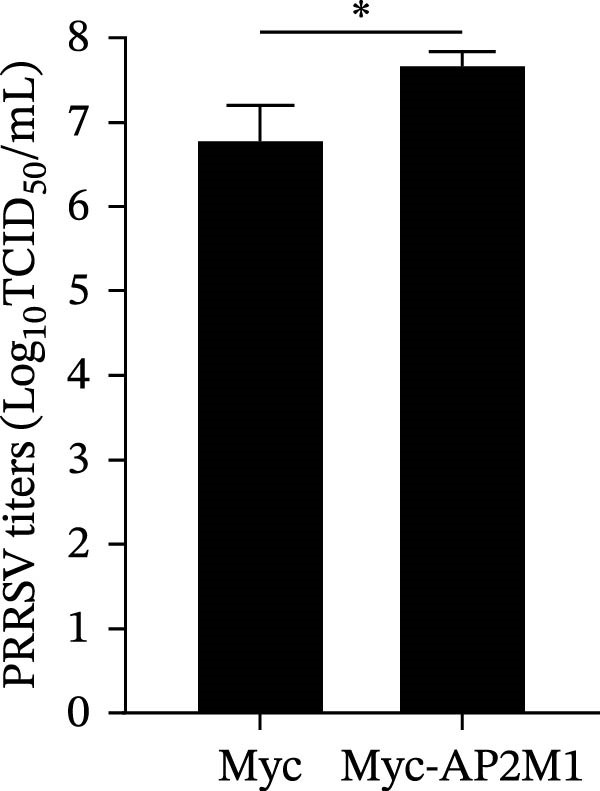
(H)

**Figure:**
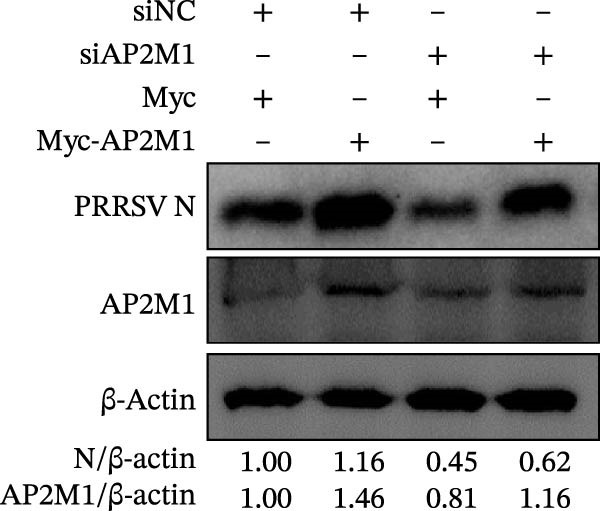
(I)

**Figure:**
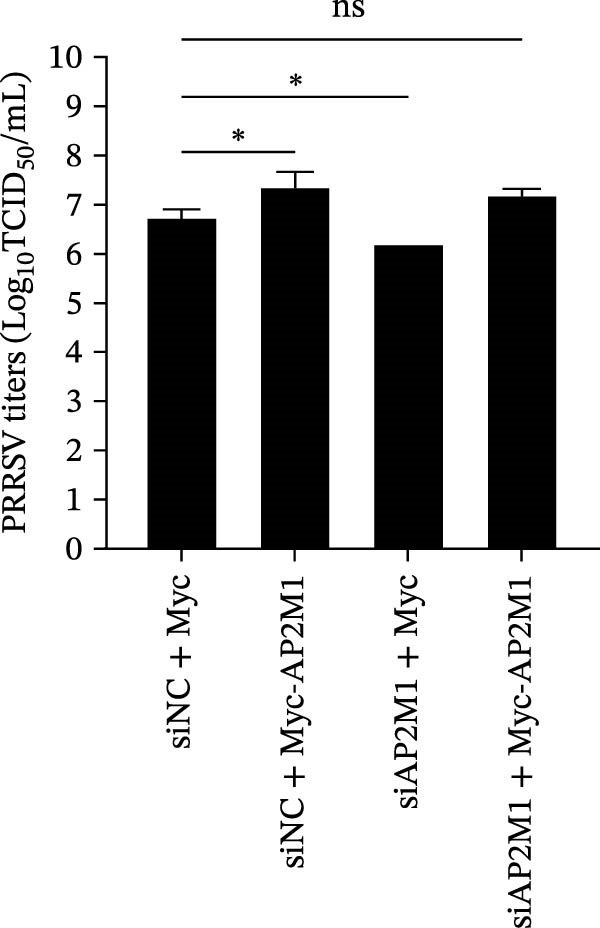
(J)

**Figure:**
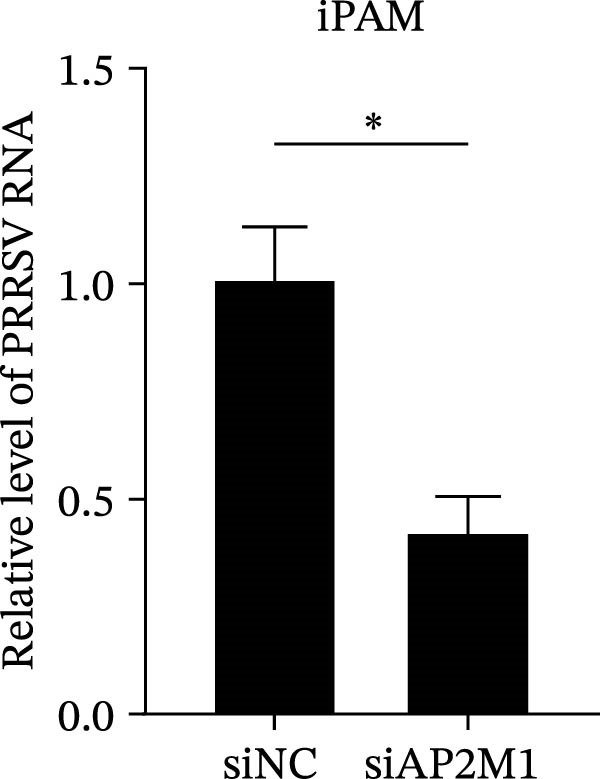
(K)

**Figure:**
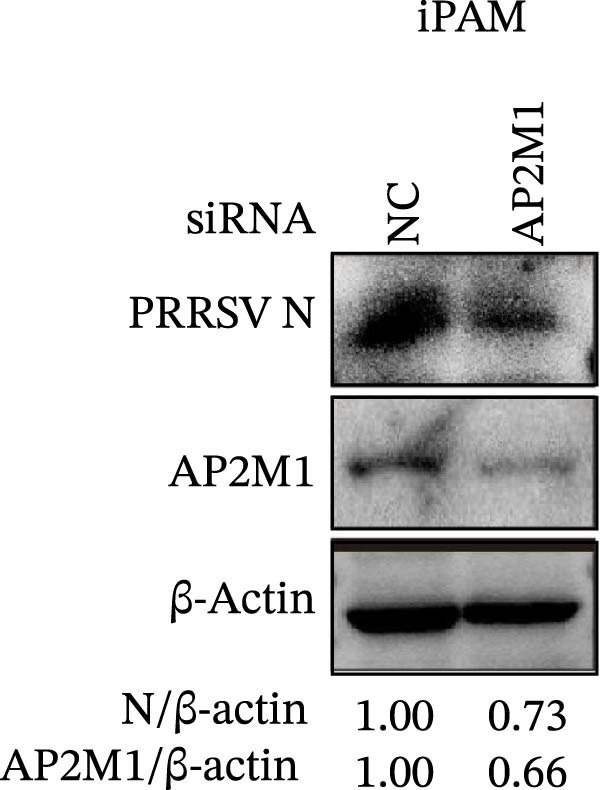
(L)

**Figure:**
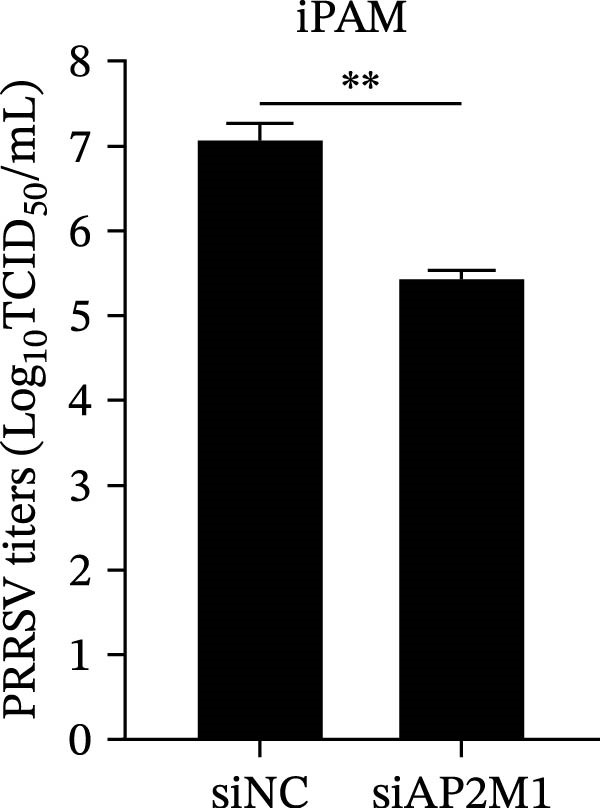
(M)

**Figure:**
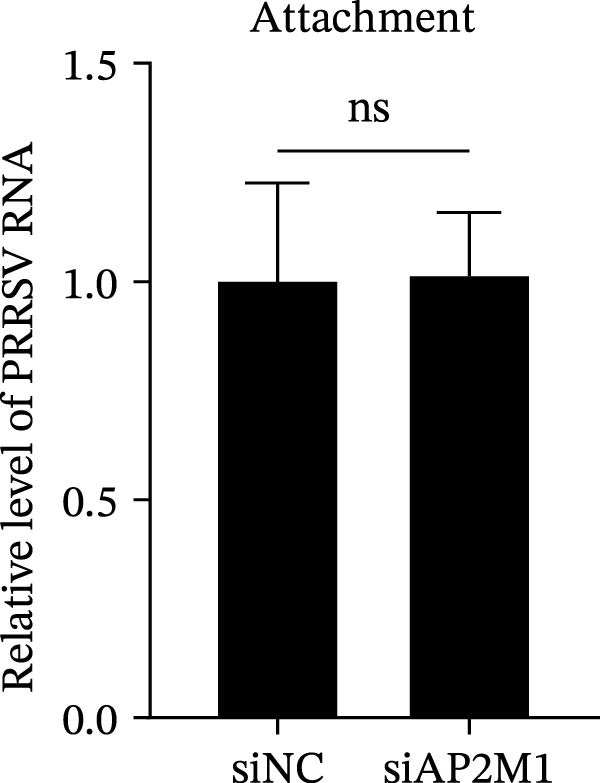
(N)

**Figure:**
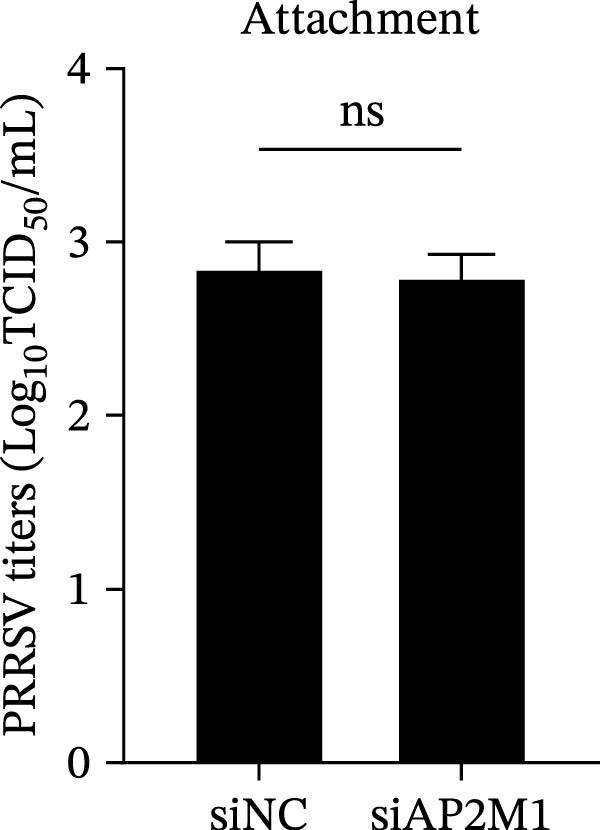
(O)

**Figure:**
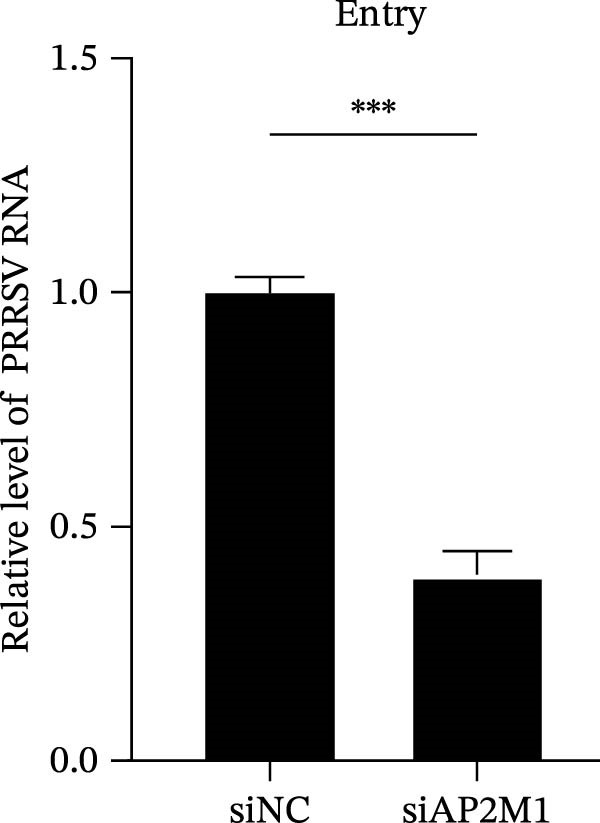
(P)

**Figure:**
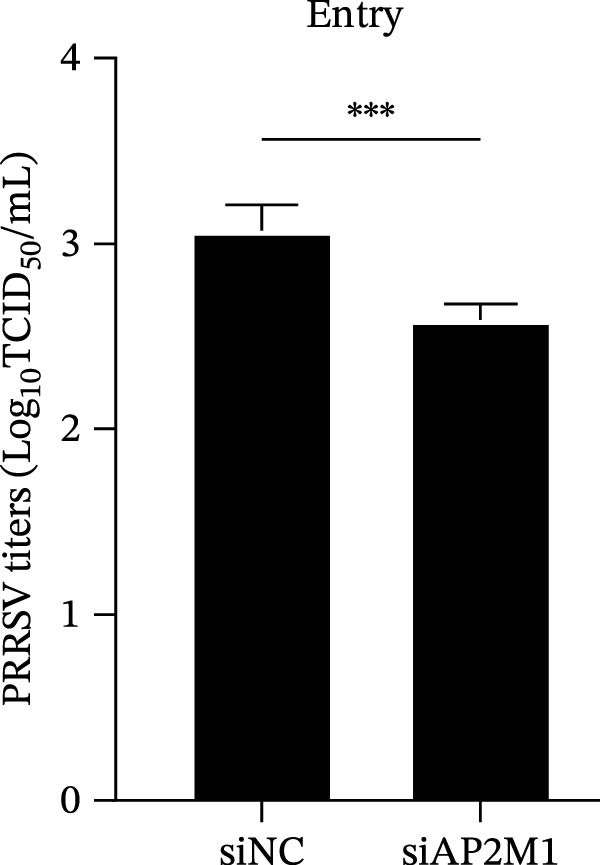
(Q)

**Figure:**
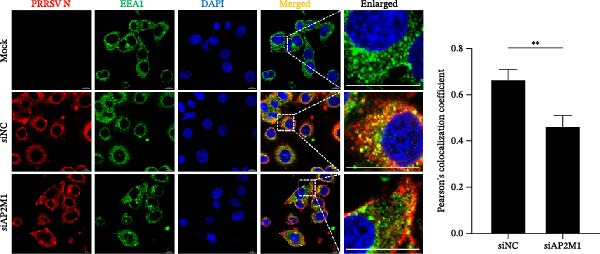
(R)

We next examined whether NAKs promoted PRRSV entry through AP2M1. RT‐qPCR and TCID_50_ assays revealed that *AP2M1* knockdown reduced internalized viral RNA abundance (60.3%; Figure 6P) and viral titers (0.5 log_10_ TCID_50_/mL; Figure 6Q), indicating impaired viral entry rather than attachment (Figure 6N,O). Furthermore, confocal microscopy showed that *AP2M1* knockdown disrupted the trafficking of internalized viral particles to EEs, with PCC decreasing from 0.66 to 0.46 (Figure 6R). Taken together, these findings establish AP2M1 as a critical downstream effector of NAKs that is required for efficient PRRSV entry.

### 3.5. Phosphorylated AP2M1 Interacts With PRRSV GP5 and Its Receptor CD163 to Facilitate Viral Entry

To elucidate the molecular mechanism by which the NAKs–AP2M1 axis mediates PRRSV entry, we next investigated the interactions between AP2M1 and both viral and host proteins. AP2M1 is known for its role in recognizing the canonical YxxØ motif within cargo proteins. Bioinformatics analysis indicated that the cytoplasmic tail of GP5 harbors a highly conserved YxxØ motif across diverse PRRSV strains (Figure 7A), suggesting that AP2M1 may facilitate viral entry through interacting with GP5. Co‐IP assays validated that exogenous Myc‐tagged AP2M1 efficiently pulled down Flag‐tagged GP5 from whole‐cell lysates (Figure 7B), and endogenous AP2M1 was specifically precipitated by Flag‐GP5 (Figure 7C). Given the critical role of CD163 as a receptor for PRRSV entry, we also explored the functional relationship between AP2M1 and CD163. Sequence alignment analysis identified a conserved YxxØ motif within the cytoplasmic tail of CD163 across multiple mammalian species (Figure 7D). Co‐IP assays further revealed that AP2M1 interacted with endogenous CD163 in both Marc145 (Figure 7E,F) and iPAM cells (Figure 7G).

Figure 7Phosphorylated AP2M1 interacts with PRRSV GP5 and its receptor CD163 for viral entry. (A) Multiple sequence alignment of the cytoplasmic tail of GP5 from representative PRRSV strains was performed using ESPript 3.2 to identify conserved YxxØ motifs. Conserved residues are highlighted. (B) The HEK‐293T cells were co‐expressed with Myc‐AP2M1 and Flag‐GP5, Myc‐AP2M1 and empty vector (Flag), or Myc and Flag‐GP5. The cells were lysed, co‐incubated with BeyoMag protein G beads and rabbit anti‐Myc pAbs, and the interacting proteins were detected from the immunocomplex by WB. (C) The Marc145 cells expressing Flag‐GP5 or Flag were lysed, co‐incubated with BeyoMag protein G beads and mouse anti‐Flag MAb, and the endogenous AP2M1 was detected from the immunocomplex by WB. (D) Multiple sequence alignment of CD163 from different species was performed using ESPript 3.2 to identify conserved YxxØ motifs within the cytoplasmic tail. (E) The Marc145 cells expressing Myc‐AP2M1 or Myc were lysed, co‐incubated with BeyoMag protein G beads and rabbit anti‐Myc pAbs, and the endogenous CD163 was detected from the immunocomplex by WB. The Marc145 (F) or iPAM (G) cells were lysed, co‐incubated with BeyoMag protein G beads and mouse anti‐Myc MAb, and the endogenous AP2M1 was detected from the immunocomplex by WB. (H) The HEK‐293T cells were co‐expressed with mCherry‐GP5, EGFP‐AP2M1, and Flag‐CD163 for 24 h. Cells were then fixed, and the subcellular localization of these proteins was determined by confocal microscopy. Representative images are shown. Scale bars = 20 μm. The colocalization between these proteins was assessed by determining the PCC using the ImageJ software. (I) The HEK‐293T cells were co‐expressed with EGFP‐AP2M1 and mCherry‐GP5, or EGFP‐AP2M1[T156A] and mCherry‐GP5. Cells were then fixed, and the subcellular localization of these proteins was determined by confocal microscopy. The colocalization was assessed by determining the PCC using the ImageJ software. (J) The HEK‐293T cells were co‐expressed with EGFP‐AP2M1 and Flag‐CD163, or EGFP‐AP2M1[T156A] and Flag‐CD163. Cells were then fixed, and the subcellular localization of these proteins was determined by confocal microscopy. The colocalization was assessed by determining the PCC using the ImageJ software. Data represent means ± SEM from three independent experiments. Statistical analysis was carried out using the Student’s *t*‐test.  ^∗∗^ *p* < 0.01.

**Figure:**
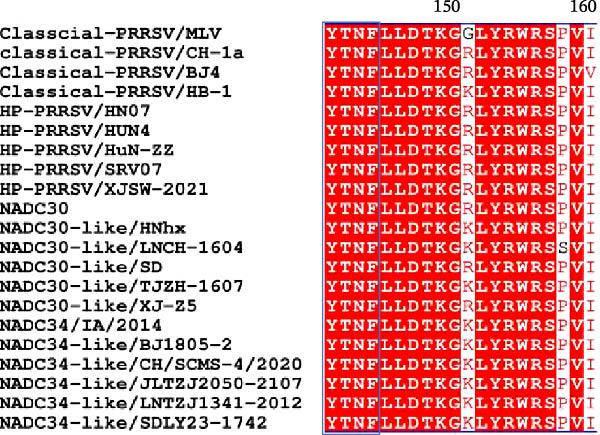
(A)

**Figure:**
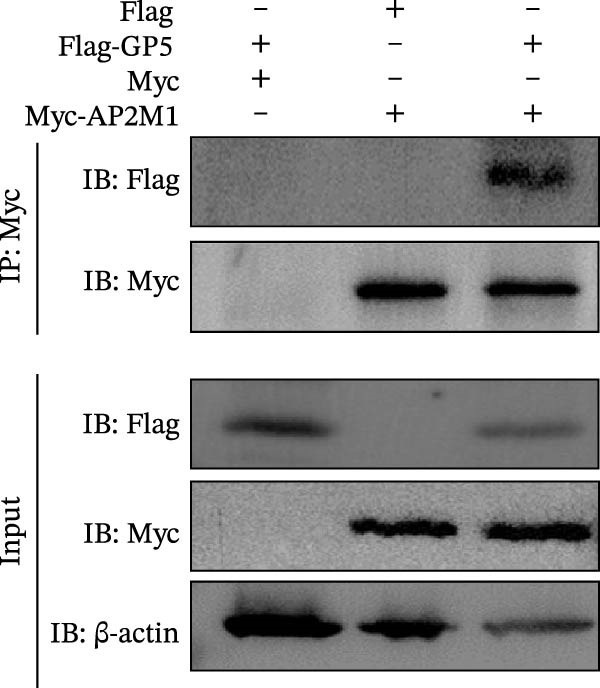
(B)

**Figure:**
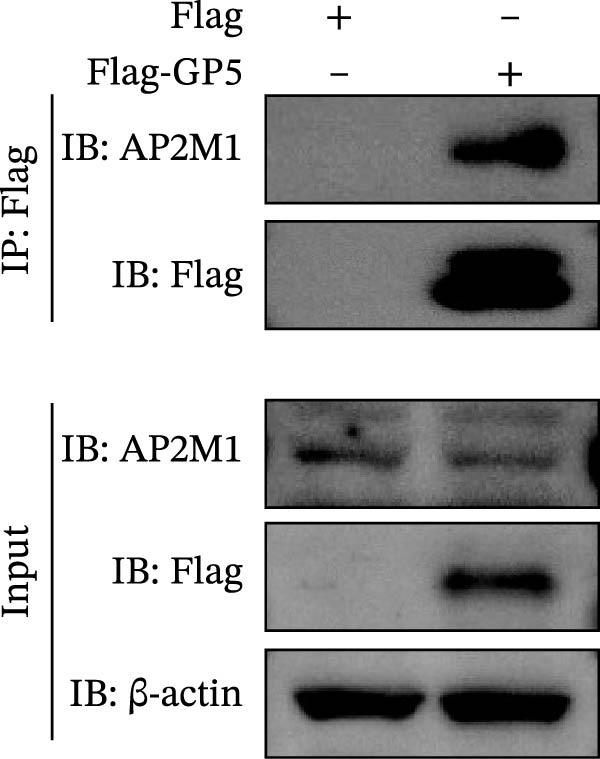
(C)

**Figure:**
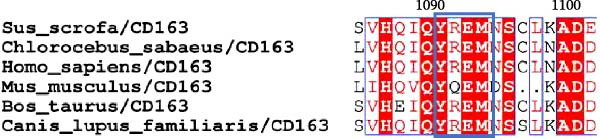
(D)

**Figure:**
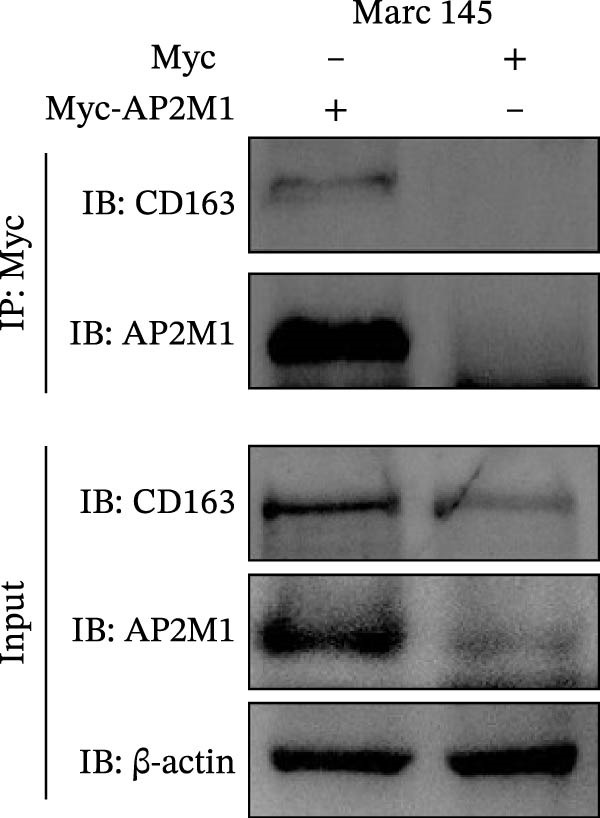
(E)

**Figure:**
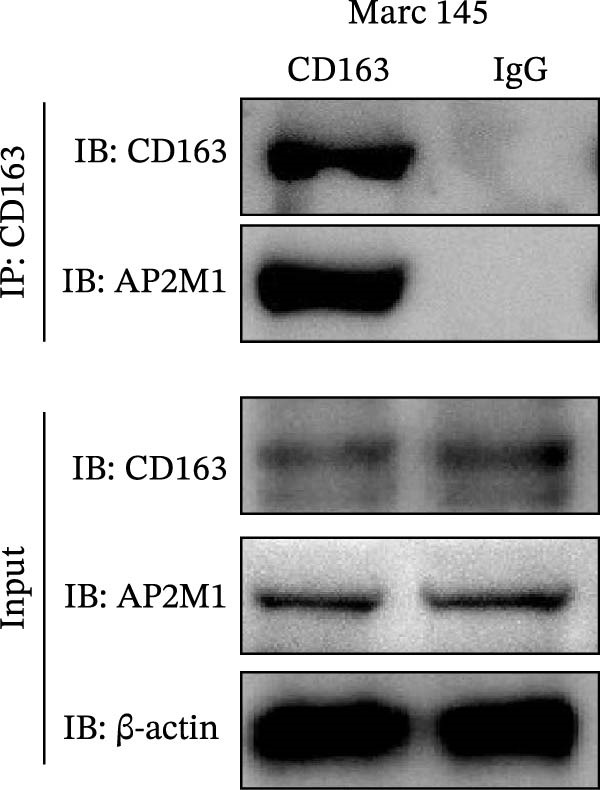
(F)

**Figure:**
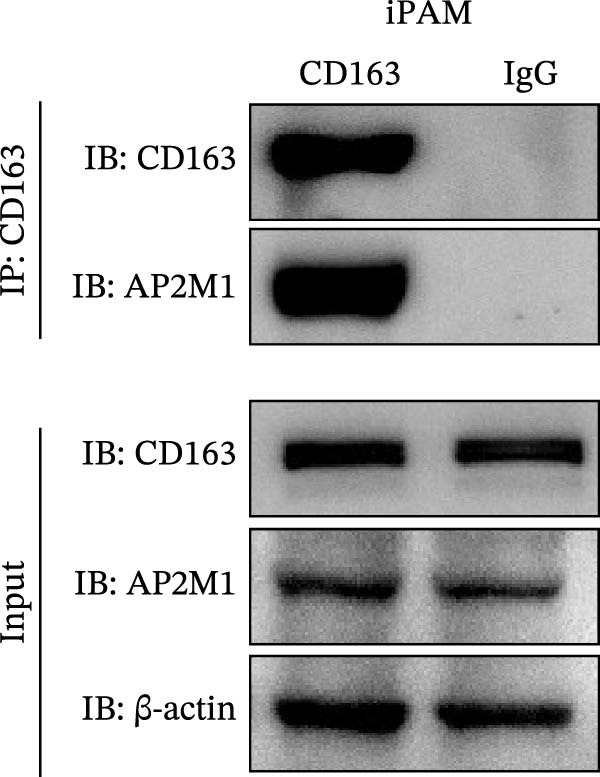
(G)

**Figure:**
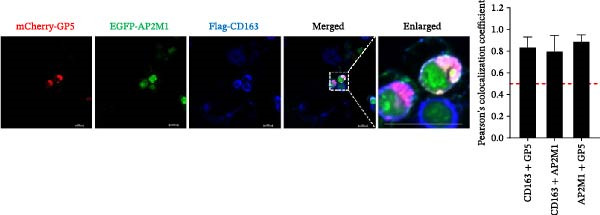
(H)

**Figure:**
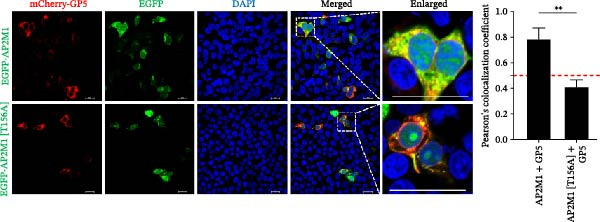
(I)

**Figure:**
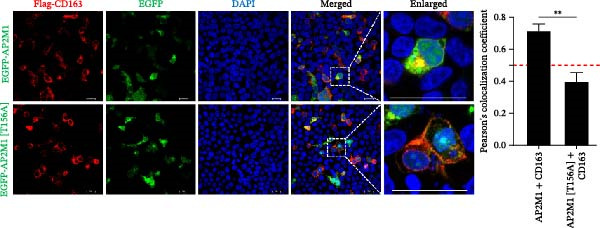
(J)

Based on the interactions between AP2M1 and GP5, AP2M1 and CD163, together with previously reported GP5–CD163 interactions [41, 42], we hypothesized that AP2M1 may act as a molecular adaptor to form a heterotrimeric complex with GP5 and CD163. To test this hypothesis, triple‐fluorescence confocal microscopy was employed to examine their subcellular localization. Figure 7H demonstrated a strong colocalization among AP2M1, GP5, and CD163 (PCC >0.5), suggesting that they collaboratively form a functional complex to facilitate PRRSV entry.

To further evaluate the importance of AP2M1 phosphorylation in its interactions with GP5 and CD163, the HEK‐293T cells were co‐expressed mCherry‐GP5 or Flag‐CD163 with either wild‐type (wt) AP2M1 (EGFP–AP2M1) or mutant AP2M1 (EGFP–AP2M1[T156A]), a phosphorylation‐deficient mutant in which threonine 156 was substituted with alanine. Confocal microscopy revealed that in the presence of wtAP2M1, both GP5 and CD163 were diffusely distributed throughout the cytoplasm, and the wtAP2M1 exhibited significant colocalization with GP5 (PCC = 0.78; Figure 7I, top panel) and CD163 (PCC = 0.74; Figure 7J, top panel). In contrast, the colocalization of AP2M1[T156A] with GP5 (PCC = 0.41; Figure 7I, bottom panel) and CD163 (PCC = 0.40; Figure 7J, bottom panel) were markedly reduced, as characterized by the punctate distribution of AP2M1[T156A] within the cytoplasm, and the aggregation of GP5 and CD163 at or near the cell membrane region.

Collectively, these findings indicate that phosphorylated AP2M1 interacts with PRRSV GP5 and its receptor CD163 to form a functional complex, thereby facilitating efficient viral entry.

### 3.6. AP2M1–YxxØ Interaction Is Required for PRRSV Entry

To assess whether the interactions between AP2M1 and CD163 or GP5 depend on the YxxØ motif, we employed the small‐molecule compound ACA, which competitively binds the hydrophobic pocket of AP2M1 and disrupts its interaction with the YxxØ motif within cargo proteins without affecting AP2M1 phosphorylation [26]. Initially, the noncytotoxic concentrations of ACA in Marc145 cells were determined to be ≤2.50 μM, with 1.50 or 2.00 μM selected for subsequent experiments (Figure 8A). RT‐qPCR, WB, and virus titration assays indicated that ACA suppressed PRRSV infection at indicated concentrations, as evidenced by reduced in viral RNA levels (42.1% and 46.8%; Figure 8B), N protein expression (Figure 8C), and infectious virus titers (0.7 and 0.8 log_10_ TCID_50_/mL; Figure 8D). In line with these results, IFA showed that ACA reduced PRRSV infectivity by 26.8% and 26.4% (Figure 8E). Moreover, RT‐qPCR and confocal microscopy showed that ACA impaired PRRSV entry, as indicated by decreased viral RNA levels (51.1%; Figure 8F) and reduced colocalization of PRRSV N protein with EEs (PCC decreased from 0.63 to 0.39; Figure 8G). Notably, ACA significantly disrupted the colocalization of AP2M1 with both CD163 and GP5 (PCC <0.5) without affecting CD163–GP5 interaction (PCC = 0.81; Figure 8H). Collectively, these findings suggest that the AP2M1–YxxØ interaction is crucial for PRRSV entry.

Figure 8AP2M1–YxxØ interaction is required for PRRSV entry. (A) The Marc145 cells were treated with increasing concentrations of ACA for 72 h, and the cytotoxicity was evaluated using the CCK‐8 assay. Cell viability in DMSO‐treated cells was normalized to 100%. (B–E) The Marc145 cells were pretreated with ACA for 2 h and then co‐incubated with PRRSV (MOI = 0.1) and the inhibitor for 24 h. PRRSV infection was evaluated by determining the intracellular viral RNA abundance using RT‐qPCR (B), viral N protein expression using WB (C), extracellular viral titers using TCID_50_ assay (D), and viral infectivity by IFA with staining of the viral N protein (PRRSV N: green; nuclei: blue; scale bars: 50 μm) (E). The viral infectivity was calculated as the percentage of viral N protein‐positive cells relative to total cells. At least ten images were analyzed from each of the three independent experiments. Representative images are shown. (F, G) The ACA‐ or D MSO‐pretreated Marc145 cells were infected or not infected with PRRSV (MOI = 5) at 4°C for 1 h. The cells were washed to remove the unbound viral particles, and then shifted to 37°C for 1 h. PRRSV entry was assessed by determining internalized viral RNA abundance using RT‐qPCR (F) and the subcellular localization of viral N protein with EEA1 by confocal microscopy (G). Representative confocal images are shown. Scale bars = 10 μm. The colocalization was assessed by determining the PCC using the ImageJ software. (H) The ACA‐ or DMSO‐pretreated HEK‐293T cells were co‐expressed with mCherry‐GP5, EGFP‐AP2M1, and Flag‐CD163 for 24 h. Cells were then fixed, and the subcellular localization of these proteins was determined by confocal microscopy. The colocalization was assessed by determining the PCC using the ImageJ software. Data represent means ± SEM from three independent experiments. Statistical analysis was carried out using the one‐way ANOVA or Student’s *t*‐test.  ^∗^ *p* < 0.05;  ^∗∗^ *p* < 0.01;  ^∗∗∗^ *p* < 0.001; ns, not significant, *p* > 0.05.

**Figure:**
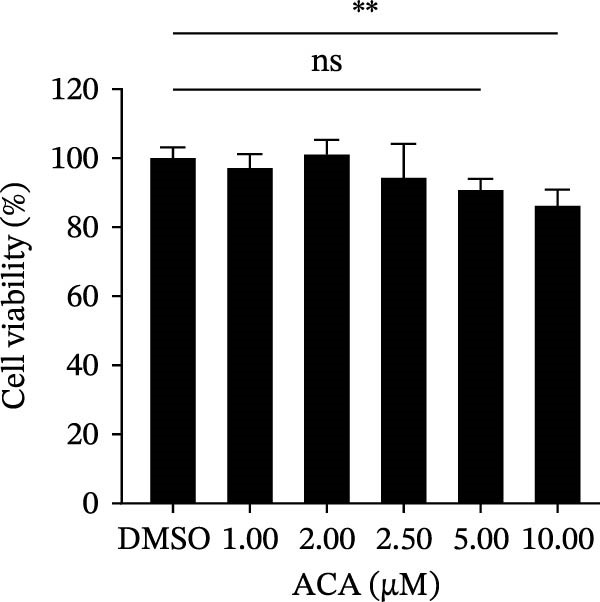
(A)

**Figure:**
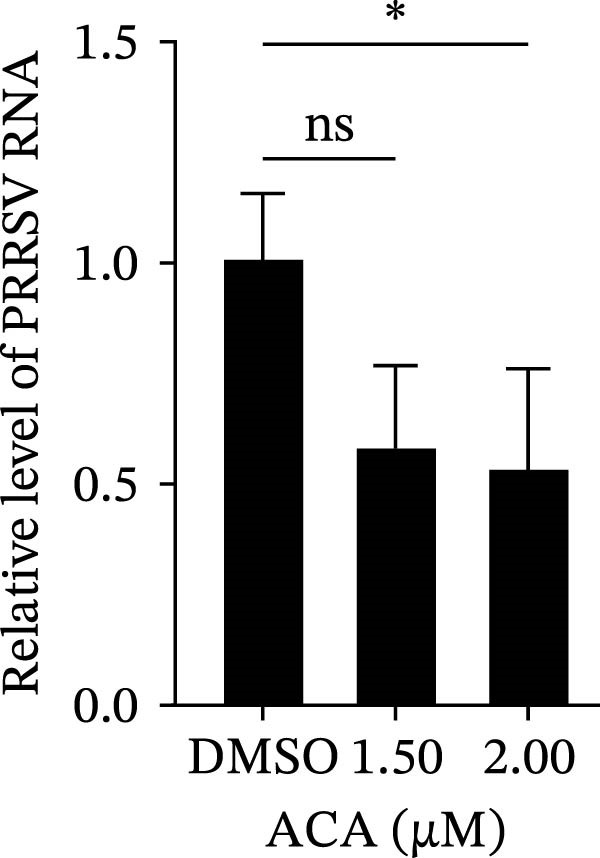
(B)

**Figure:**
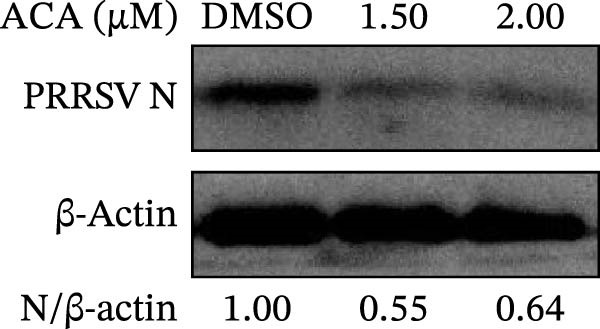
(C)

**Figure:**
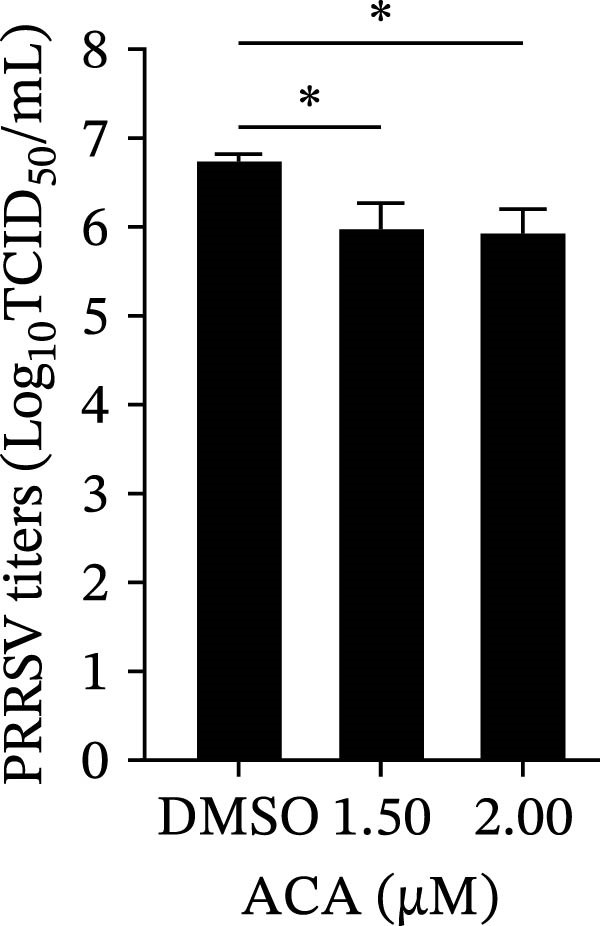
(D)

**Figure:**
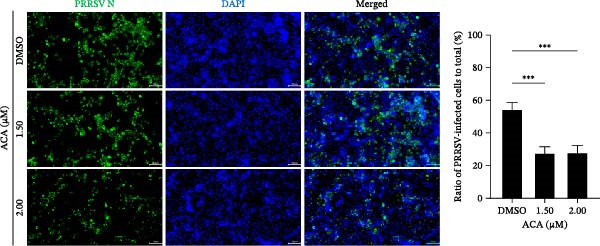
(E)

**Figure:**
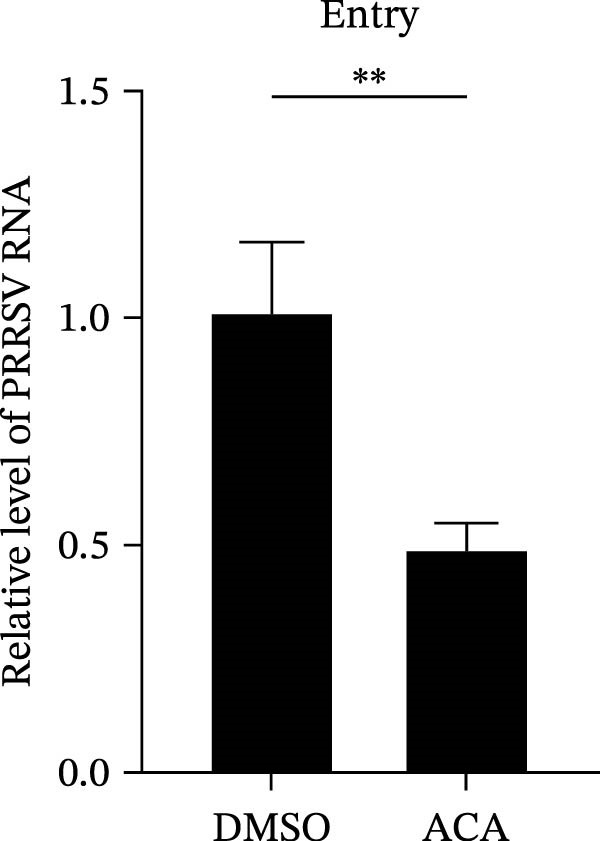
(F)

**Figure:**
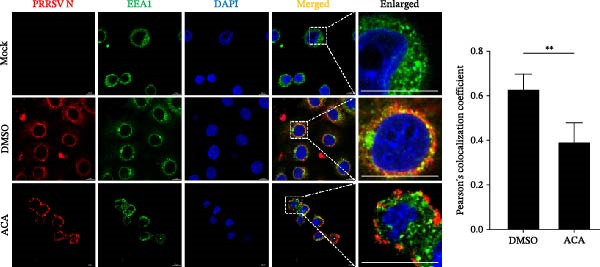
(G)

**Figure:**
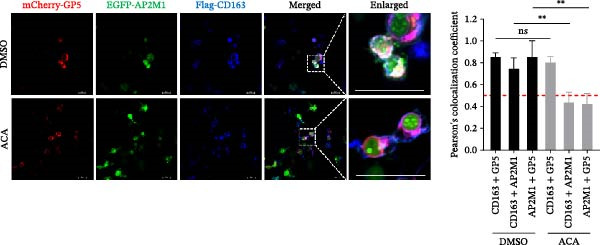
(H)

### 3.7. NAKs Serve as Broad‐Spectrum Anti‐PRRSV Targets

To evaluate whether the NAKs–AP2M1 axis could be exerted as a broad‐spectrum antiviral target against PRRSV, we investigated its roles in the infection of diverse viral strains. Inhibitor assays revealed that sunitinib effectively suppressed PRRSV infection across multiple strains, including the highly pathogenic HN07‐1 and HuN4, NADC30‐like strain HNhx, and VR2332‐derived MLV strain, as demonstrated by a reduction in viral RNA levels (28.6%–64.4%; Figure 9A) and viral titers (0.6–1.4 log_10_ TCID_50_/mL; Figure 9B). Furthermore, depletion of individual NAKs did not affect PRRSV attachment (Figure 9C,D) but consistently hindered viral entry across all tested strains, resulting in decreased internalized viral RNA levels (37.7%–63.6%; Figure 9G) and viral titers (0.3–0.7 log_10_ TCID_50_/mL; Figure 9H). Collectively, these findings suggest that the NAKs–AP2M1 axis is broadly required for efficient PRRSV entry, underscoring its potential as a broad‐spectrum antiviral target against PRRSV.

Figure 9NAKs serve as broad‐spectrum anti‐PRRSV targets. (A, B) The Marc145 cells were pretreated with sunitinib for 1 h, then co‐inoculated with the different PRRSV strains (HN07‐1, HuN4, HNhx, or MLV; MOI = 0.1) and the inhibitor at 37°C for 24 h. The antiviral activity of sunitinib was evaluated by determining the intracellular viral RNA abundance using RT‐qPCR (A), and extracellular viral titers using TCID_50_ assay (B). (C, D) The siRNA‐transfected Marc145 cells were infected with different PRRSV strains (MOI = 5) at 4°C for 1 h. After washing with ice‐cold PBS, PRRSV attachment was evaluated by detecting the cell‐bound viral RNA abundance using RT‐qPCR (C) or viral titers using TCID_50_ assay (D). (E, F) The siRNA‐transfected Marc145 cells were infected with PRRSV (MOI = 5) at 4°C for 1 h. After washing away the unbound virions, the cells were shifted to 37°C for 1 h to allow virus internalization. PRRSV entry was assessed by detecting the internalized viral RNA abundance using RT‐qPCR (E) or viral titers using TCID_50_ assay (F). Data represent means ± SEM from three independent experiments. Statistical analysis was carried out using the one‐way ANOVA or Student’s *t*‐test.  ^∗^ *p* < 0.05;  ^∗∗^ *p* < 0.01;  ^∗∗∗^ *p* < 0.001;  ^∗∗∗∗^ *p* < 0.0001; ns, not significant, *p* > 0.05.

**Figure:**
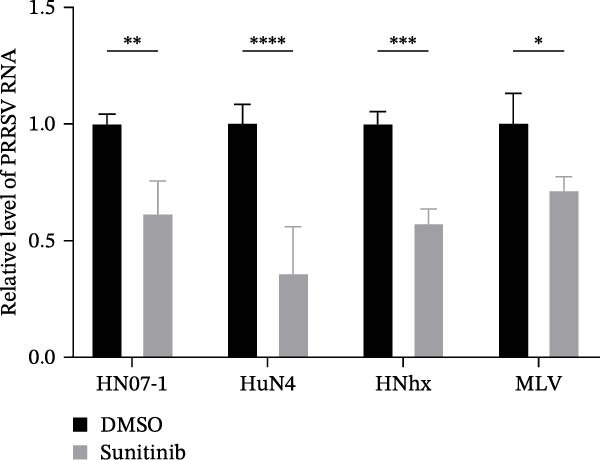
(A)

**Figure:**
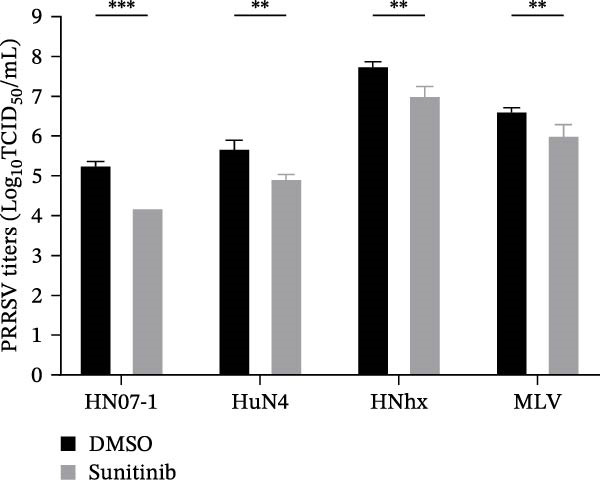
(B)

**Figure:**
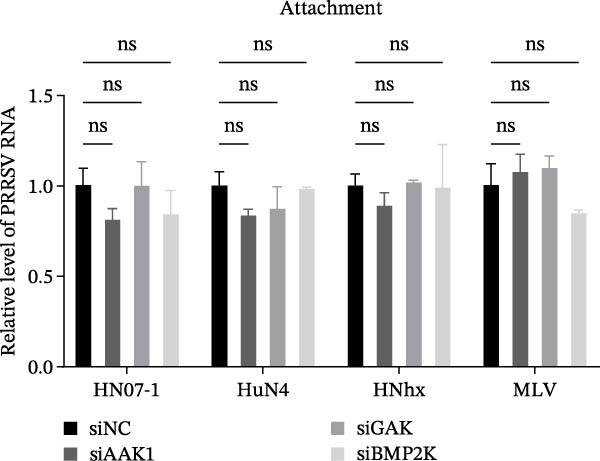
(C)

**Figure:**
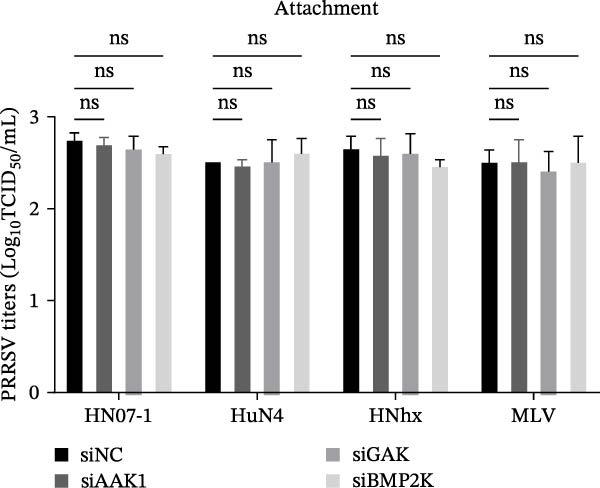
(D)

**Figure:**
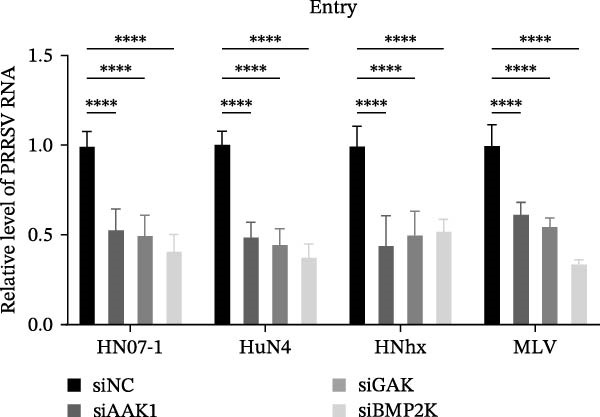
(E)

**Figure:**
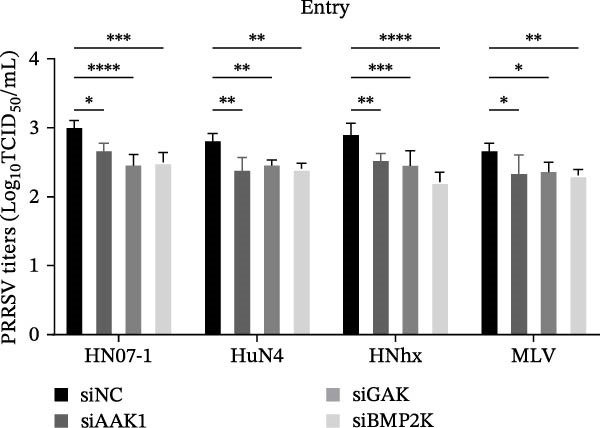
(F)

## 4. Discussion

PRRSV is a major pathogen that poses a significant threat to the global swine industry. The mechanism of its entry, particularly the internalization facilitated by the coordinated action of viral envelope proteins and the receptor CD163, has been a longstanding focus of research [43–45], while the host regulatory factors involved in this process remain inadequately understood [46, 47]. In this study, we identified, for the first time, the crucial roles of the NAK members AAK1, GAK, and BMP2K in PRRSV entry.

We initially conducted an siRNA‐based screen targeting four NAK family members and confirmed that PRRSV selectively exploited AAK1, GAK, and BMP2K, but not STK16, to support viral infection (Figure 1). Our finding aligns with previous research on other viruses, such that dengue virus (DENV) exploits AAK1, GAK, BMP2K, rather than STK16 [48], while severe acute respiratory syndrome coronavirus 2 (SARS‐CoV‐2) exploits all four NAK family members [31]. In contrast, Toscana virus (TOSV) utilizes only AAK1 and GAK [30]. This selectivity may be attributed to species‐ or cell type‐specific differences in STK16 expression or sequence/structural variations in its AP2M1‐binding region that affect binding affinity. The precise mechanism by which NAKs are selectively utilized by PRRSV warrants further investigation. We further demonstrated that the NAK inhibitors sunitinib and erlotinib also effectively inhibited PRRSV infection (Figure 2).

We then sought to characterize the stages of the PRRSV life cycle targeted by NAKs. Through knockdown and pharmacological inhibitor experiments, we established that NAKs predominantly contributed to PRRSV entry and, to a lesser extent, its release, with no significant effects on viral attachment, replication, or assembly (Figures 3 and 4). Consistent with our findings, previous studies have identified the involvement of AAK1 in the entry of rabies virus (RABV), DENV, hepatitis C virus (HCV) [33, 49, 50], GAK in the entry of HCV and TOSV [30, 32], and BMP2K in the entry of DENV [48]. Given the established role of NAKs in post‐Golgi vesicular trafficking and membrane dynamics [18], their inhibition may subtly disrupt vesicle maturation or transport events required for efficient virion egress, thereby leading to a modest reduction in PRRSV release. Additionally, the NAK inhibitors sunitinib and erlotinib have been shown to impede the release of SARS‐CoV‐2 and DENV by disrupting NAK kinase activity [31, 50], which further supports our speculation.

As protein kinases, NAKs exhibit the capacity to bind to a wide range of substrate proteins. Notably, the NAK family members AAK1, GAK, and BMP2K can phosphorylate the AP2M1 at T156 residue, thereby facilitating cargo endocytosis via the CME pathway [22, 51–53]. Our study corroborated that NAKs can phosphorylate AP2M1 in Marc145 cells (Figure 5). Additionally, we verified that AP2M1 was integral to transport the PRRSV particles to the EEs during viral entry (Figure 6). Notably, previous research has indicated that certain viruses also exploit the NAKs–AP2M1 axis for viral entry, such as TOSV, HCV, DENV, and SARS‐CoV‐2 [32, 33, 48, 54].

To elucidate the specific mechanism by which AP2M1 facilitated PRRSV entry, we performed Co‐IP assays, revealing that AP2M1 interacted with PRRSV GP5 and its receptor CD163 (Figure 7A–G). In addition, confocal microscopy demonstrated colocalization of AP2M1, GP5, and CD163, with weakened colocalization between AP2M1 and both GP5 and CD163 when the phosphorylation site of AP2M1 was mutated (Figure 7H–J). These results suggested that AP2M1 participated in PRRSV entry, contingent upon its phosphorylation. Importantly, disruption of the AP2M1–YxxØ interaction by the compound ACA impeded PRRSV entry, indicating that AP2M1 contributed to PRRSV entry by interacting with the YxxØ motifs in both CD163 and GP5 (Figure 8). Finally, we demonstrated that the NAKs–AP2M1 axis could serve as a potential broad‐spectrum anti‐PRRSV drug target (Figure 9).

Previous studies have established that CD163 is not involved in PRRSV attachment, but is crucial for viral uncoating within the EEs during entry [10, 11, 55, 56]. GP5 is required for PRRSV entry through its interaction with CD163 [41, 42] or MYH9 [57–60]. AP2M1 is well‐known for its roles in modulating the formation of clathrin‐coated pits and linking the membrane‐localized receptors containing the YxxØ motif to clathrin for CME initiation, as well as mediating the recycling of the intracellular receptors to the cell membrane [61, 62]. Notably, CD163 contains a conserved YxxØ motif within its cytoplasmic tail, and the canonical model indicates that CD163 undergoes CME to release its ligand within endosomes, after which it is recycled back to the plasma membrane or trafficked to the lysosome for degradation [63, 64]. Disruption of intracellular trafficking or lysosomal function has been shown to alter CD163 turnover [65], supporting the possibility that AP2M1 dysfunction may impair the proper membrane trafficking itinerary of CD163, thereby leading to lysosomal degradation rather than its normal recycling. Alternatively, phosphorylated AP2M1 may interact with the dissociated PRRSV GP5, which is then incorporated into the clathrin‐coated vesicles for CME‐dependent entry. AP2M1 may also facilitate the delivery of the membrane‐localized CD163 into the EEs for efficient membrane fusion and uncoating during PRRSV entry. Consistent with our study, AP2M1 is involved in the CME‐dependent entry of SARS‑CoV‑2, HCV, human papillomavirus, and pseudorabies virus via binding to the YxxØ motif within viral envelope proteins or receptors [32, 66–69].

In conclusion, our study highlights a previously unrecognized fundamental role of the NAKs–AP2M1 axis in the CME‐dependent entry of PRRSV. These findings provide new insights into virus–host interactions and identify this axis as a potential target for the development of broad‐spectrum antiviral strategies.

## Author Contributions

**Longxiang Zhang:** conceptualization, writing, funding acquisition, review, revision. **Rui Li:** conceptualization, data collection, writing, revision. **Yan Jiang and Xinrong Wang:** original draft preparation, formal analysis. **Junhai Zhu and Nan Yan:** methodology, software. **Yue Wang:** supervision, funding acquisition, review, revision.

## Funding

This work was supported by grants from the National Natural Science Foundation of China (Grants 32302852 and 32573404), the Natural Science Foundation of Chongqing (Grant CSTB2023NSCQ‐MSX0366), the Fundamental Research Funds for the Central Universities (Grant SWU‐KQ22035), and the Science and Technology Research Program of Chongqing Municipal Education Commission (Grant KJQN202300224).

## Ethics Statement

This study did not involve any animal experiments. Therefore, ethical approval for animal use was not required.

## Conflicts of Interest

The authors declare no conflicts of interest.

## Data Availability

The data that support the findings of this study are available from the corresponding author upon reasonable request.
